# Integrating resource utilization and bioregenerative life support systems for sustainable space exploration

**DOI:** 10.3389/fmicb.2026.1837116

**Published:** 2026-05-26

**Authors:** Mattia Esposito, Luca Tonietti, Rosa Santomartino, Donato Giovannelli, Elena Chianese, Alessandra Rotundi, Ida Romano, Annarita Poli, Ilaria Finore, Angelina Cordone, Paola Di Donato

**Affiliations:** 1 Department of Environmental Sciences, Informatics and Statistics, University of Venice Ca' Foscari, Venice, Italy; 2 INAF-OACN, Osservatorio Astronomico di Capodimonte, Naples, Italy; 3 Institute of Biomolecular Chemistry, National Research Council, CNR-ICB, Pozzuoli, Italy; 4 CNR-IPCF, Institute for Chemical and Physical Processes, National Research Council, Messina, Italy; 5 Department of Science and Technology, University Parthenope, Naples, Italy; 6 Department of Biological and Environmental Engineering, Cornell University, Ithaca, NY, United States; 7 Department of Biology, University of Naples Federico II, Naples, Italy; 8 National Research Council, Institute of Marine Biological Resources and Biotechnologies, CNR-IRBIM, Ancona, Italy; 9 Department of Marine and Coastal Science, Rutgers University, New Brunswick, NJ, United States; 10 Woods Hole Oceanographic Institution, Department of Marine Chemistry and Geochemistry, Woods Hole, MA, United States; 11 Earth-Life Science Institute, Tokyo Institute of Technology, Tokyo, Japan

**Keywords:** astrobiology, *in situ* resource utilization, bioregenerative life support systems, space biotechnology, biomining, extremophiles, closed-loop life support, space exploration

## Abstract

The long-term presence of humans in space depends on reducing reliance on Earth's resupply of materials and resources. *In situ* resource utilization (ISRU) represents a sustainable approach to support human activities in space by converting local materials into consumables, propellants, and structural feedstocks. In parallel, bioregenerative life support systems (BLSS) primarily sustain internal loop closure (LC) by regenerating air, H_2_O, nutrients, and food from habitat-contained streams. Traditional ISRU concepts have primarily focused on abiotic technologies that process rocks, regolith, and atmospheric components to extract O_2_, H_2_O, and metals, although biotic approaches are also under investigation. Biological and bio-hybrid approaches, guided by microorganisms and other living systems, could complement these technologies by supporting both external resource conversion and internal LC in future space exploration. In this review, we adopt a resource-centric framework to compare abiotic, biotic, and coupled resources acquisition pathways across the main functional domains relevant to both ISRU and BLSS, treated as operationally distinct but architecturally coupled subsystems within a broader resource-management framework. We discuss the main functional domains required for human settlement, spanning external resource conversion (O_2_, CO_2_, H_2_, CH_4_, H_2_O, materials, manufacturing, energy) and internal regenerative functions (food production, air and H_2_O revitalization, and waste recycling). For each domain, we describe representative abiotic (e.g., MOXIE, ROXY, molten-regolith electrolysis, fission surface power, and advanced solar arrays) and biotic systems, for both ISRU-relevant bioprocesses (e.g., biomining, biopolymer production) and BLSS components (e.g., plant-growth and microbial recycling loops). The most realistic path toward sustainable human settlements beyond Earth orbit lies in coupled ISRU-BLSS architectures in which external resource acquisition and internal regenerative loops are coordinated across shared material and energy flows.

## 1 Introduction

In crewed space exploration missions, payloads represent the most prohibitive aspect, both logistically and economically, with costs reaching tens of thousands of dollars per kilogram (Jones, 2018; Averesch et al., 2023). Advances such as reusable rockets and more efficient propulsion systems aim to reduce these costs, but the full range of resources required for long-term crewed missions extends well beyond propellant. Human crews will require food, H_2_O, O_2_, consumables, and other commodities. Under current mission concepts, these needs are met through payloads launched from Earth, with estimated masses averaging 5 t for a 180-day lunar mission and 14 t for a 540-day Martian mission (Averesch et al., 2023). This makes our dependence on Earth resources a critical limitation that needs to be solved to unlock sustainable long-term crewed missions (Coopersmith, 2011; Kornuta et al., 2019; Elsaesser et al., 2023; Puumala et al., 2023; Kim, 2025).

The potential use of extraterrestrial resources to sustain extended crewed missions emerged in the mid-20th century as a promising solution (Ehricke, 1979; Duke et al., 1985). In the 1950s, Arthur C. Clarke speculated that lunar-derived propellants could significantly enhance the feasibility of deep-space missions by reducing dependence on Earth-based launches (Clarke, 1951; Spudis, 1999). By the 1960s, governmental reports and technical analysis began exploring the broader implications of space resource utilization (Carr, 1963; Cilliers et al., 2020). The conceptualization of Project Horizon, a proposed lunar base, introduced the idea of utilizing *in situ* materials for mission sustainability, ideally using lunar regolith as a natural shield against radiation and micrometeoroid impacts, and repurposing empty propellant tanks for storage applications (Ordway et al., 1988). *In situ* resource utilization (ISRU) strategies emerged as a pathway for sustaining human presence beyond Earth while reducing the logistical constraints of interplanetary travel (Meurisse and Carpenter, 2020; Starr and Muscatello, 2020; Hoffman et al., 2022; Guerrero-Gonzalez and Zabel, 2023). At present, ISRU refers to different strategies that aim to utilize, transform, and repurpose materials present on planetary bodies (Sanders, 2000; Sanders et al., 2008; Anand et al., 2012; Pajares et al., 2025). Depending on the specific location (e.g., the Moon, Mars, asteroids), resources available for ISRU include mineral solids (e.g., regolith, rocks), volatile and atmospheric components (H_2_O ice, volatiles, atmospheric and trapped gases), which could provide oxidants, fuels, H_2_O, and chemical feedstocks (Starr and Muscatello, 2020; Hoffman et al., 2022; Cilliers et al., 2023; Santomartino et al., 2023; Robinot et al., 2025). To date, most development has focused on abiotic ISRU, proposing pathways to utilize regolith as a source of O_2_ and metals via high-temperature reduction, molten-salt or molten-regolith electrolysis (MSE and MRE) and related gas-solid reactions (Yan and Fray, 2010; Xie et al., 2017; Lomax et al., 2022; Guerrero-Gonzalez and Zabel, 2023; Burke et al., 2024). However, advancements in biotechnologies have opened emerging microbial and bioengineering approaches for ISRU, potentially enabling resource extraction and *in situ* biomanufacturing (Srichandan et al., 2019; Cockell et al., 2020; Kaksonen et al., 2021; Berliner et al., 2022; Cockell and Santomartino, 2022; Arif et al., 2023; Tonietti et al., 2024). Microbial-mediated regolith mining represents a promising biological ISRU strategy that could offer a safer and more sustainable approach to metal extraction compared to traditional chemical processes, potentially reducing risks to equipment and crew exposure to hazardous compounds (Cockell et al., 2021; Gumulya et al., 2022; Santomartino et al., 2022, 2026; Tonietti et al., 2023).

In addition to metals and gases, which could be extracted from the local environment, long-duration missions will require systems capable of producing or regenerating air, H_2_O, nutrients, and food within the habitat. These functions are typically addressed by bioregenerative life support systems (BLSS), which use primarily biological processes to sustain closed-loop resource regeneration.

Microorganisms, microalgae, and higher plants can perform numerous functions, including fixing CO_2_, generating O_2_ and other gases (e.g., H_2_ and CH_4_), producing edible biomass and reduced carbon compounds, mobilizing metals from regolith, and converting organic and inorganic waste streams into reusable resources (Slenzka and Kempf, 2010; Billi et al., 2013; Massa et al., 2020; Poughon et al., 2021; De Micco et al., 2023). Among these biological platforms, phototrophic microorganisms are especially relevant to coupled ISRU-BLSS architectures because they can link CO_2_ fixation, O_2_ regeneration, edible biomass production, nutrient recovery, and the conversion of ISRU-derived or recycled inputs within compact photobioreactor modules (Verseux et al., 2016; Fahrion et al., 2021; Revellame et al., 2024; Ellena et al., 2024). Portions of BLSS have been tested on the ISS, demonstrating their potential for atmospheric revitalization, water treatment, and food production through coupled microbial and plant-based modules (Bartsev et al., 1996; De Micco et al., 2023; Kane and Shasteen, 2024; Liistro et al., 2024).

**Figure 1:**
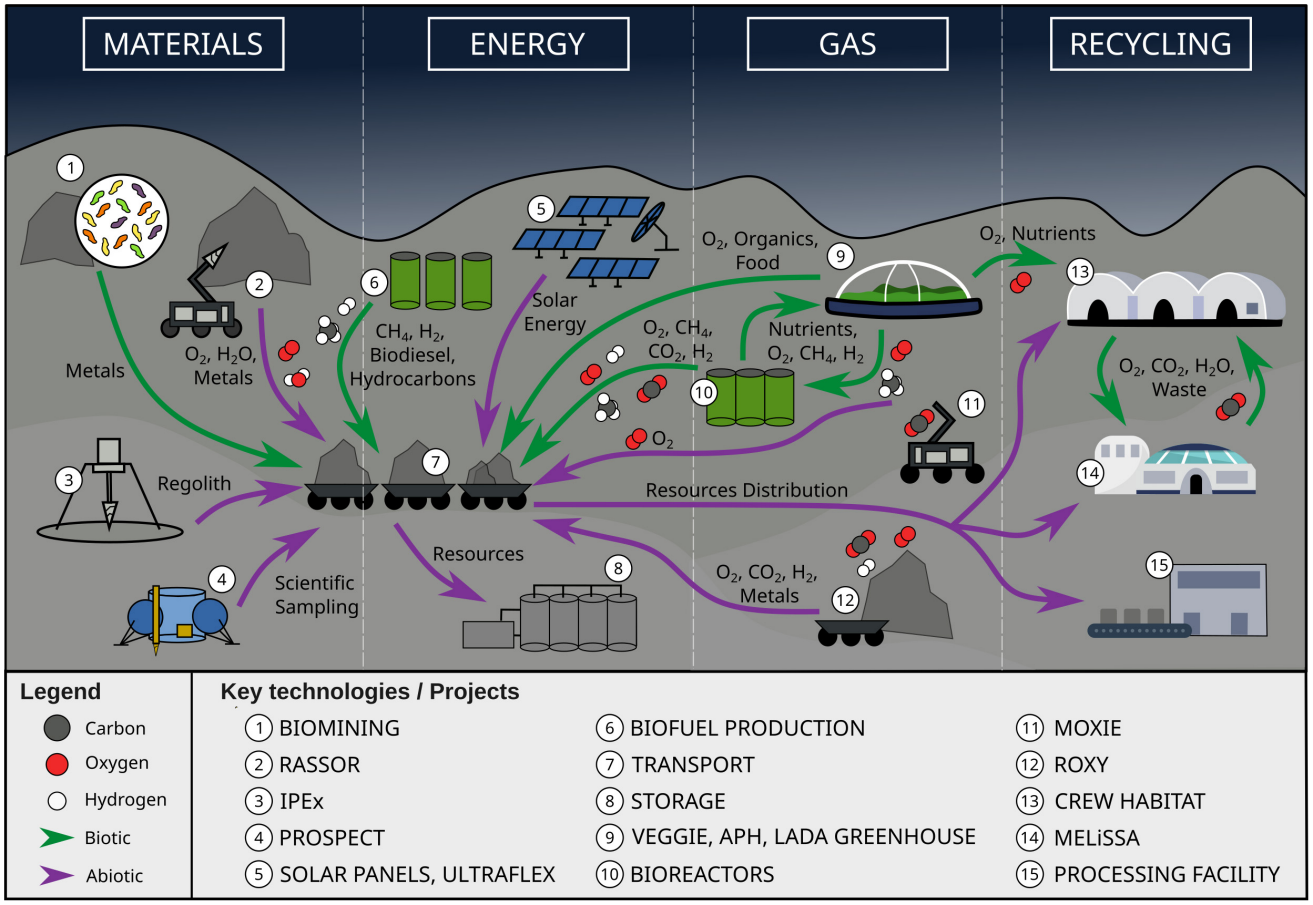
Schematic representation of coupled *in situ* resource utilization (ISRU) and bioregenerative life support system (BLSS) functions within a long-duration exploration architecture. The diagram illustrates interconnected material, energy, gas, and recycling loops relevant to sustained human presence beyond LEO. Numbers indicate specific technologies and, where available, projects or experiments demonstrating the corresponding processes. Regolith and atmospheric CO_2_ are processed through biomining, chemical reduction (e.g., MOXIE and ROXY), and biofuel production to generate O_2_, CH_4_, H_2_, metals, hydrocarbons, and other resources. Solar panels provide primary power for extraction, storage, and processing systems. Biological modules, including microbial bioreactors, algae containers, and greenhouses, support food production, nutrient cycling, and atmospheric regeneration. Outputs are distributed to crew habitats, vehicles, and processing facilities, forming a resource-management architecture that maximizes recycling while reducing dependence on Earth resupply.

Each of these abiotic and biotic processes offers promising capabilities for ISRU or BLSS, but no individual pathway can satisfy the combined resource demands of future crewed settlements. Sustained exploration will require architectures that couple external resource acquisition with internal recovery, regeneration, and reuse, thereby reducing dependence on Earth-supplied payloads while maintaining operational resilience (Figure 1). Although recent studies increasingly recognize the need to couple ISRU and BLSS for sustained exploration, abiotic resource extraction, biological resource utilization, and bioregenerative life support are still commonly treated through partially separate technological frameworks (Starr and Muscatello, 2020; Meurisse and Carpenter, 2020; Verseux et al., 2022; De Micco et al., 2023; Kane and Shasteen, 2024). In line with the mission-class framework proposed by Averesch et al. (2023), which relates mission classes to *in situ* resource availability and logistic resupply, long-duration missions are expected to increasingly rely on the progressive interaction between ISRU and loop closure (LC). In the present review, this classification provides a structural reference to examine how ISRU and BLSS functions interact across resource domains. ISRU is primarily associated with the acquisition and conversion of resources from the surrounding planetary environment. In parallel, BLSS supports internal LC through the recovery, regeneration, and biological transformation of resources already circulating within the habitat. These domains remain functionally distinct, yet their interaction becomes increasingly important as exploration shifts from short-duration missions toward sustained presence. Here, we adopt a resource-oriented framework to analyze their potential integration. For each key resource required for long-duration missions (e.g., O_2_, CO_2_, H_2_, CH_4_, materials, food, and energy), we examine the available pathways, considering their roles in external resource utilization and their contribution to internal regenerative functions. This perspective allows ISRU and BLSS to be analyzed as distinct but tightly coupled subsystems within a broader resource-management architecture.

The literature discussed in this narrative review was identified through searches of major scientific databases, including Scopus, Google Scholar, and PubMed, using combinations of keywords related to ISRU, BLSS, astrobiology, space biomanufacturing, and space resource utilization. Priority was given to recent and highly cited studies, complemented by seminal contributions relevant to the development of ISRU, BLSS, and their potential integration.

## 2 Functional organization of ISRU and BLSS

To analyze how ISRU and BLSS could interact, resource utilization can be framed in terms of the main operational stages required to access, transform, and manage extraterrestrial materials and the resource flows linking external utilization to internal regenerative functions.

ISRU can be achieved by abiotic and biotic approaches. Abiotic ISRU relies on purely physicochemical operations (e.g., thermal extraction, gas-solid reactions, high-temperature reduction, electrolysis, sintering) to convert regolith, ice, and atmospheric volatiles into oxidants, propellants, structural materials, and other consumables (Anand et al., 2012; Anderson et al., 2021; Cao et al., 2023; Guerrero-Gonzalez and Zabel, 2023). Biotic ISRU employs microorganisms to mobilize elements from extraterrestrial substrates and to drive bioprocessing routes for resource extraction or *in situ* biomanufacturing (Gumulya et al., 2022; Santomartino et al., 2023). In contrast, the recovery, regeneration, and conversion of resources already circulating within the habitat are functions of BLSS rather than ISRU. These processes include air and H_2_O revitalization, production of edible biomass, and transformation of internal waste streams, within systems that involve almost exclusively biotic processes (Bartsev et al., 1996; Slenzka and Kempf, 2010; Revellame et al., 2024). Together, these approaches represent a continuum, ranging from purely abiotic systems, through processes assisted by biological steps, to fully coupled architectures in which biological and abiotic modules operate on the same resources.

The technological maturity of these approaches, expressed here as technology readiness level (TRL) according to NASA standards, is heterogeneous. Some ISRU systems have already been demonstrated in space. The Mars Oxygen *in situ* Resource Utilization Experiment (MOXIE) validated O_2_ production from atmospheric CO_2_ on Mars, and biomining experiments such as BioRock and BioAsteroid proved that microorganisms can leach metals from rocks under extraterrestrial conditions, although scale-up to industrial standards has not been demonstrated yet (Cockell et al., 2020, 2021; Hecht et al., 2021; Hoffman et al., 2022; McClean et al., 2022; Santomartino et al., 2024, 2026). Numerous BLSS components have also operated on the ISS, including plant-growth systems such as the Vegetable Production System (VEGGIE), the Advanced Plant Habitat (APH), and LADA Greenhouse (Levinskikh et al., 2005; Massa et al., 2020; Bunchek et al., 2021). By contrast, other technologies remain at intermediate to low TRLs. These include molten-regolith electrolysis concepts (e.g., Regolith to Oxygen and Metal Conversion, ROXY; Molten Salt Electrolysis and Metallothermic Reduction Integration, MOSARI), large-scale regolith excavation systems (e.g., Regolith Advanced Surface Systems Operations Robot, RASSOR; ISRU Pilot Excavator, IPEx), and fully closed BLSS loops such as the Micro-Ecological Life Support System Alternative (MELiSSA), all of which have so far been validated only in laboratories or terrestrial analog environments (Hendrickx et al., 2006; Gumulya et al., 2022; Santomartino et al., 2023; Seidel et al., 2023; Robinot et al., 2025).

The utilization of resources in space faces different problems: (i) reservoir identification (RID), (ii) extraction (EX), (iii) transport (TR), (iv) processing and conversion (PR), and (v) storage and stabilization (ST). RID refers to the detection and characterization of accessible material in the environment (Byrne, 2009; Cilliers et al., 2020; Starr and Muscatello, 2020). EX is the physical, chemical, or biological detachment of the target resource from its original matrix (Just et al., 2019; Sowers et al., 2022). TR consists of moving solid and fluid resources across the system, including transport from external ISRU operations to processing modules and distribution within habitats. In integrated architectures, TR therefore represents a key interface between ISRU modules accessing external reservoirs and BLSS subsystems managing internal resource circulation (Just et al., 2019; Chung et al., 2021; Cloud et al., 2021; Simonini et al., 2024). For biological systems, TR additionally includes circulation of culture media, gas exchange, and biomass harvesting under conditions of low gravity, temperature extremes, vacuum or low pressure in which conventional terrestrial mechanisms no longer hold (Huang et al., 2018; Adam et al., 2020). PR are the operations that transform extracted inputs into different products. In space, these operations must function with limited power, minimal crew intervention, and high reliability, while facing dust constraints and potentially corrosive or toxic compounds (Starr and Muscatello, 2020; Cilliers et al., 2023). ST are required to ensure that products remain usable over relevant timescales, without structural degradation, contamination, or phase changes (Kinefuchi et al., 2022; Simonini et al., 2024). Biological stocks (e.g., microorganisms, seeds, spores) must retain viability under space conditions. Thus, the choice of EX and PR pathways is strongly linked to feasible ST options.

Framing resource utilization in space in terms of these generic problems (RID, EX, TR, PR, ST) highlights that abiotic and biotic strategies address overlapping but not identical bottlenecks (Table 1). Abiotic systems are most effective in EX and PR for high-throughput, high-temperature transformations. Biotic systems primarily contribute to PR and stabilization processes and can also support LC and recycling functions at the interface between ISRU modules and BLSS subsystems, particularly when processing heterogeneous feedstocks and waste streams, as emphasized in recent roadmaps on microbial applications for sustainable space exploration (Averesch et al., 2023; Koehle et al., 2023; Santomartino et al., 2023). In this framework, RID, EX, TR, PR, and ST organize the main operational stages through which resources are identified, accessed, transformed, and managed. Because throughput, energy demand, and operating conditions depend strongly on the specific resource and technological implementation, these stages are treated here primarily as functional categories rather than as quantitative classes.

**Table 1:** Summary of representative technologies and processes investigated to obtain mission-relevant resources.

Mission Requirement	Primary Sources	Type	Representative techniques and demonstrators	RID	EX	TR	PR	ST	Application Scale
O_2_	Regolith, Ice, CO_2_	A, B, H	MOXIE, ROXY, MOSARI, Bacteria, DOP	yes	yes	yes	yes	yes	L, G
H_2_O	Ice, Hydrated Minerals	A, B, H	Thermal Extraction	yes	yes	yes	yes	yes	L, G
Gasses	Volatiles	A, B, H	CO_2_ capture, MSE, electrolysis	yes	yes	yes	yes	yes	L, G
Propellant	H_2_, CH_4_, LOX	A, B, H	LOX from regolith, Sabatier	yes	yes	yes	yes	yes	G
Energy	Solar, isotopes	A, B, H	FSP, UltraFlex, Sabatier, Biofuels	yes	yes	yes	yes	yes	L, G
Materials	Regolith	A, B, H	RASSOR, IPEx, PROSPECT, Biomining	yes	yes	yes	yes	yes	L, G
Manufacturing	Feedstock, imported	A, B, H	Biomining, sintering	no	no	yes	yes	yes	L, G
Food	Bioregenerative systems	B, H	VEGGIE, APH, LADA	yes	yes	yes	yes	yes	L
Recycling	Waste	A, B, H	MELiSSA	no	no	yes	yes	yes	L, G
Storage	Cross-cutting function	A, H	Cryotanks, pressure vessels	no	no	yes	yes	yes	L, G
Health	H_2_O, habitat consumable, shielding	A, B, H	Environmental Control, BLSS, sterilization	yes	yes	yes	yes	yes	L
Communication	Energy, infrastructure	A	Antenna, power systems	no	no	yes	yes	yes	G

Abiotic (A), biotic (B), and hybrid (H) possible implementations are indicated. The table summarizes primary resource sources, representative techniques and technology demonstrators, and their coverage of reservoir identification (RID), extraction (EX), transport (TR), processing (PR), and storage/stabilization (ST). Listed projects and experiments (e.g., MOXIE, MELiSSA, VEGGIE, RASSOR) are included as representative implementations of the corresponding functional domains and techniques, rather than as exhaustive or one-to-one correspondences. For each process, a local (L) or global (G) applicability scale is indicated, where local (L) refers to module or site-specific operations, and global (G) refers to functions with habitat or system-level relevance for resource circulation and management. Abbreviations: LOX, liquid oxygen; MSE, molten salt electrolysis; DOP, dark oxygen production; FSP, fission surface power; BLSS, bioregenerative life support system; APH, Advanced plant habitat; LADA, Lada greenhouse; RASSOR, Regolith Advanced Surface Systems Operations Robot; IPEx, *In situ* Polar Ice Extraction; MOSARI, Modular Oxygen Storage and Regolith Integration; PROSPECT, Package for Resource Observation and *in situ* Prospecting for Exploration, Commercial exploitation and Transportation; MELiSSA, Micro-Ecological Life Support System Alternative. The table is intended as a qualitative comparative framework and does not provide quantitative performance metrics across resource classes.

## 3 Pathways for resource acquisition and regeneration

Biological systems offer promising opportunities for long-term space missions by enabling the extraction and transformation of extraterrestrial resources into O_2_ and other strategic gases (e.g., CO_2_, H_2_, and CH_4_), materials, and chemical feedstocks, while also supporting regenerative functions in closed-loop habitats (Gòdia et al., 2002; Billi et al., 2021; Keller et al., 2021; Poughon et al., 2021; Mapstone et al., 2022; Billi, 2026). Microorganisms can drive multi-step processes, such as biomining and biomanufacturing of polymers and catalysts (Averesch et al., 2023). Bioleaching and weathering contribute to the extraction of metals and nutrients from regolith and chemically complex substrates that are difficult to handle with purely abiotic operations (Cockell et al., 2020; Santomartino et al., 2022; Tonietti et al., 2024). Metabolic conversion in photobioreactors and fermenters transforms CO_2_ and C and N-bearing compounds into O_2_, H_2_O, CH_4_, H_2_, and edible or processable biomass to be consumed, stored, or further transformed (Verseux et al., 2016; Detrell, 2021). Abiotic ISRU strategies and technologies rely on physical and chemical processes to transform local planetary resources. These pathways include O_2_ production from metal oxides in regolith, H_2_ generation via electrolysis, CO_2_ capture from planetary atmospheres, and CO_2_ methanation via the Sabatier reaction to produce CH_4_ and H_2_O (Anand et al., 2012; Muscatello and Santiago-Maldonado, 2012; Just et al., 2019; Vogt et al., 2019; Hecht et al., 2021). Abiotic systems can be engineered for high-temperature and high-throughput operations and are particularly suited to the extraction, concentration, and transformation of local materials (Starr and Muscatello, 2020; Radl et al., 2023; Seidel et al., 2023; Robinot et al., 2025). In the following subsections, we examine the main resources required for long-duration missions and the potential biotic, abiotic, and coupled pathways enabling their extraction, generation, or transformation.

### 3.1 Oxygen

O_2_ is essential for sustaining human activity beyond Earth, and it can be supplied through complementary pathways with different operational roles. Abiotic ISRU primarily addresses the extraction of O_2_ from extraterrestrial reservoirs through processes such as H_2_O electrolysis, photocatalytic or photolytic splitting of volatiles, and high-temperature reduction of metal oxides. On Mars, O_2_ can also be generated directly from atmospheric CO_2_ by electrochemical dissociation, as demonstrated by NASA's MOXIE experiment aboard the Perseverance rover (Hecht et al., 2021; Hoffman et al., 2022). In parallel, molten salt electrolysis systems such as ESA's ROXY process and related metallothermic-electrochemical approaches enable direct O_2_ liberation from regolith-derived oxides while yielding metallic byproducts (Seidel et al., 2022, 2023). Biological systems contribute mainly within BLSS, where photosynthetic organisms produce O_2_ from CO_2_ and H_2_O while also sustaining atmospheric revitalization through coupled carbon fixation, biomass generation, food production, and waste recycling (Gòdia et al., 2002; Mapstone et al., 2022). Biological pathways are viewed as complementary to abiotic ISRU rather than as direct substitutes for high-throughput extraction technologies. Their contribution depends on reactor productivity, available volume, illumination, and integration with broader habitat loops. Photosynthetic organisms, e.g., cyanobacteria, microalgae, and higher plants, represent the most well-studied biological O_2_ sources, capable of converting CO_2_ and H_2_O into O_2_ via the general reaction (R1) (Fahrion et al., 2021; Keller et al., 2021; De Micco et al., 2023).


$$\begin{array}{l} 6\text{C}{\text{O}}_{2}+6{\text{H}}_{2}\text{O}+\text{h}\text{ν}\to {\text{C}}_{6}{\text{H}}_{12}{\text{O}}_{6}+6{\text{O}}_{2}\;\text{(R1)} \end{array}$$

Recently, putative dark oxygen production (DOP) pathways, including microbially mediated O_2_ release under anoxic conditions, have broadened the conceptual range of possible biological O_2_ production mechanisms, sustained also in the absence of light (Ettwig et al., 2012; Ruff et al., 2023). Microbiological modules are core components of BLSS and can interface directly with ISRU-derived resources, although their practical contribution depends strongly on process configuration and mission context (Gòdia et al., 2002; Poughon et al., 2021).

#### 3.1.1 Photosynthesis

Photosynthetic microorganisms constitute a primary focus due to their structure, fast growth rates, and adaptability to closed photobioreactor environments (De Micco et al., 2023; Sukhinov et al., 2023). Reported performances vary strongly with strain and photoreactor configuration, but representative values indicate that biological O_2_ production is quantifiable at the reactor scale. For *Limnospira indica* PCC8005, biomass productivity up to 0.58 g L^−1^ d^−1^ was reported in a 90 L airlift photobioreactor at 932 μmol photons m^−2^ s^−1^, with maximum O_2_ productivity reaching 1.19 mmol O_2_ L^−1^ h^−1^ (0.038 g O_2_ L^−1^ h^−1^) (Garcia-Gragera et al., 2021). At this maximum reported productivity, approximately 158 L of active culture volume would be required to match 6 g O_2_ h^−1^, and approximately 316 L to match 12 g O_2_ h^−1^, corresponding to the minimum design rate and theoretical maximum output reported for MOXIE (Hecht et al., 2021; Hoffman et al., 2022; Garcia-Gragera et al., 2021). In *Chlorella vulgaris*, biomass productivity up to 1.29 g L^−1^ d^−1^ and biomass concentrations up to 12.24 g L^−1^ have been reported in a microgravity-capable membrane raceway photobioreactor (Helisch et al., 2020). These values highlight the potential of photobiological modules, but also their strong dependence on irradiance, reactor design, gas-liquid mass transfer, and sustained culture performance. Cyanobacteria (e.g., *Chroococcidiopsis, Synechocystis, Limnospira*, and *Arthrospira* genera) remain the most studied option, as they perform oxygenic photosynthesis with high efficiency and elevated resilience to stressors such as radiation, desiccation, and variable light regimes (Baqué et al., 2013a,b, 2016; Verseux et al., 2016; Billi, 2019, 2026). This process in species such as *Chlorogloeopsis fritschii* and *Synechocystis* sp. can operate under simulated light spectra characteristic of M-dwarf stars, expanding the range of stellar spectral conditions under which biological O_2_ production may be feasible (Battistuzzi et al., 2023a,b). Microalgae (e.g., *Chlorella sorokiniana* and *Chlamydomonas reinhardtii*) offer similar efficient light-to-biomass conversion and can be cultivated at high densities to produce O_2_ in compact volumes (Mapstone et al., 2022; Revellame et al., 2024).

#### 3.1.2 Dark oxygen production (DOP)

DOP refers to the biological or chemical production of O_2_ in the absence of sunlight (Ettwig et al., 2012; Ruff et al., 2023). Some microorganisms, such as *Nitrospira marina* and *Nitrosopumilus maritimus*, have shown the ability to produce O_2_ through oxidation of nitrogen compounds in dark and anoxic environments, e.g., deep-sea sediments, oxygen minimum zones, or underground ecosystems (Kraft et al., 2022; Ruff et al., 2023). However, the recent proposal of DOP generation in deep-seafloor environments remains under active debate, and both the reported measurements and their mechanistic interpretation are still contested (Denny et al., 2024; Downes et al., 2024; Sweetman et al., 2024). DOP, as a space-relevant O_2_ production concept, remains highly speculative. Should the existence of dark oxygen production in the deep seafloor be confirmed, it would expand our current understanding of microbial metabolism and biogeochemical cycling of elements with potential implications for planetary sciences.

#### 3.1.3 Mars oxygen in-situ resource utilization experiment (MOXIE)

MOXIE is a technological demonstration engineered to assess the feasibility of O_2_ production from the Martian atmosphere via solid oxide electrolysis (SOXE) (Hoffman et al., 2015, 2022; Meyen et al., 2016; Hecht et al., 2021). MOXIE operates by collecting Martian air through a scroll compressor specifically designed to function under low Martian environmental pressures and to handle the presence of atmospheric dust. The compressed gas is introduced into the SOXE unit, where it is heated to approximately 800 °C to facilitate the electrochemical reduction of CO_2_ into carbon monoxide (CO) and O_2_ according to the following reaction (R2):

2CO_2_ → 2CO+O_2_ (R2)

A regulated power supply provides up to 4.0 A to drive the electrolysis process, producing O_2_ at a rate of up to 1.2 g h^−1^ per cell, with a theoretical maximum of 10–12 g h^−1^ for the entire electrolysis assembly (Hecht et al., 2021; Hoffman et al., 2022; McClean et al., 2022). The system is designed to sustain a minimum O_2_ generation rate of 6 g h^−1^ under the environmental conditions at Jezero Crater, where atmospheric intake pressure averages around 6.1 mBar, while maintaining an O_2_ purity close to 98 % (Hecht et al., 2021). Despite the engineering challenges associated with long-duration operation in a dusty Martian environment, MOXIE has successfully validated the core principles of *in situ* O_2_ production, establishing a technological foundation for future large-scale ISRU applications (Nasr et al., 2018; McClean et al., 2020).

#### 3.1.4 Regolith to oxygen and metals conversion (ROXY)

ROXY is an advanced ISRU initiative developed by Airbus and investigated in ESA-related lunar ISRU contexts aimed at establishing a sustainable method for extracting O_2_ and metallic byproducts from lunar and Martian regolith, providing resources to support life and supplying construction materials for future extraterrestrial settlements (Seidel et al., 2022, 2023; Birch et al., 2025, 2026). ROXY utilizes a high-temperature, electrochemical reduction process that decomposes metal oxides, providing both O_2_ for life support and structural metals for manufacturing and infrastructure development (Schwandt et al., 2012; Seidel et al., 2022). The high-temperature MSE of metal oxides in regolith can be summarized by the representative ilmenite (FeTiO_3_) electrolysis (R3):

$2\text{FeTi}{\text{O}}_{3}\to 2\text{F}{\text{e}}^{2+}+2\text{T}{\text{i}}^{4+}+3{\text{O}}_{2}+12{\text{e}}^{-}$ (R3)

Reaction (R3) represents a simplified anodic half-reaction. In practice, stepwise electrochemical reduction of Fe and Ti oxides proceeds through multiple intermediate ionic species in the molten electrolyte. At the core of the ROXY process is MSE coupled to a solid oxide membrane (SOM), which operates at approximately 850 °C and selectively transports oxide ions to the anode, where high-purity O_2_ is evolved (Seidel et al., 2022, 2023; Birch et al., 2026). This overall process can be represented schematically in (R4) as:

$4\text{M}{\text{e}}^{n+}+2\text{n}{\text{O}}^{2-}\to 4\text{Me}+\text{n}{\text{O}}_{2}$ (R4)

where Me is a generic metal.

The process begins with the collection and refinement of lunar or Martian soil, which is then mixed with a molten electrolyte medium. An applied current drives oxide-ion transport to the anode, where O_2_ is evolved and collected (Li et al., 2023; Burke et al., 2024; Guan et al., 2025). The cathodic reaction results in the deposition of reduced metals, such as Fe, Ti, and Al, which can be repurposed for additive manufacturing for space (AMFs), habitat construction, or spacecraft repairs. Recent pilot-plant modeling estimates that a ROXY facility with a mass of 1172 kg could process 2310 kg regolith yr^−1^ to produce 1155 kg O_2_ yr^−1^ and an equivalent mass of mixed metallic alloy, requiring 45,243 kWh yr^−1^ of electricity, equivalent to approximately 39 kWh per kg of O_2_ produced (Birch et al., 2026). Unlike previous O_2_ extraction techniques that relied on chemical reduction with H_2_, thus requiring substantial H_2_O resources, ROXY operates as a dry process, making it particularly viable for arid environments (Seidel et al., 2023; Guan et al., 2025). However, the process remains at relatively low TRL and requires downstream separation and refinement of the mixed metallic product before use in manufacturing or construction (Birch et al., 2026).

Beyond its space applications, ROXY technology has significant terrestrial implications, particularly in sustainable metallurgy and industrial decarbonization. Its electrochemical approach eliminates carbon-based reducing agents, offering a cleaner and potentially more energy-efficient alternative to traditional metal extraction processes on Earth that rely on fossil fuel combustion (e.g., blast furnaces) (Qian et al., 2024; Rippy et al., 2024).

#### 3.1.5 Molten salt electrolysis and metallothermic reduction integration (MOSARI)

MOSARI technique represents a novel approach designed specifically for the lunar environment (Radl et al., 2023). MOSARI enables the simultaneous production of O_2_ and metal alloys directly from the Moon's regolith, while limiting waste production (Radl et al., 2023). This technique involves MSE utilizing a fluoride-based electrolyte (Vogel et al., 2017; Milicevic et al., 2018; Cvetković et al., 2020), in which the lunar regolith undergoes dissociation. O_2_ gas is generated at an inert anode, while metallic elements such as Ca, Mg, Al, and Si are deposited on the cathode. Representative reactions occurring in MOSARI are shown in (R5)–(R7). These denote overall net transformations of the coupled MSE and metallothermic steps rather than full electrochemical half-reactions or system mass balances:

Al_2_O_3_ → 2Al+3/2O_2_ (R5)

SiO_2_ → Si+O_2_ (R6)

FeTiO_3_+Ca → Fe+TiO_2_+CaO (R7)

Following MSE, a metallothermic reduction step uses electrochemically produced metals as reductants to selectively process ilmenite-rich concentrates, yielding iron-rich alloys that are suitable for constructing lunar habitats (Radl et al., 2023).

### 3.2 Other strategic gases

Biological pathways primarily act on carbon and hydrogen-bearing species, transforming CO_2_ and, in coupled architectures, organic waste streams into reduced gases, such as H_2_ and CH_4_, and into energy-rich organic compounds (Verseux et al., 2016; Angelidaki et al., 2018). In a biotic context, CO_2_ serves as the principal carbon substrate, H_2_ acts both as an intermediate and a product, and CH_4_ acts as a terminal reduced gas or component of biogas (Wang et al., 2018; Fahrion et al., 2021). In an abiotic context, CO_2_ acts as the dominant carbon feedstock, H_2_ provides reducing power and reversible energy storage, and CH_4_ serves as a fuel and baseline propellant. The relevance of these pathways is strongly dependent on planetary context. On Mars, atmospheric CO_2_ provides an abundant and directly accessible carbon reservoir. On the Moon, instead, carbon is far more limited and carbon-based fuel pathways are therefore much more constrained. Storage could follow underground geological principles currently investigated on Earth for CH_4_, CO_2_, and H_2_ (Cascone et al., 2025). These gases connect atmosphere processing, regolith reduction, power generation, and propulsion, and are best analyzed at the level of integrated cycles rather than as isolated products.

#### 3.2.1 Carbon dioxide

CO_2_ is a substrate whose assimilation is critical for biological O_2_ generation, biomass production, and the synthesis of reduced carbon compounds in photosynthetic species (Mapstone et al., 2022; Santomartino et al., 2023). Oxygenic phototrophs fix CO_2_ via the Calvin-Benson-Bassham cycle, converting inorganic carbon into carbohydrates and other organic metabolites while releasing O_2_ (McKinlay and Harwood, 2010; Hudson, 2024). Anoxygenic phototrophic bacteria and chemoautotrophs (e.g., *Moorella, Hydrogenobacter*, and *Aquifex* genera) expand the options for CO_2_ fixation pathways (e.g., reverse TCA cycle, 3-hydroxypropionate cycle, Wood–Ljungdahl pathway), allowing carbon assimilation in the absence or low light availability (Ragsdale and Pierce, 2008; Hügler and Sievert, 2011; Bar-Even et al., 2012). In coupled biological reactors interfacing with ISRU, these organisms can act as CO_2_ scrubbers that transform CO_2_ into biomass, gaseous intermediates, or extracellular products (e.g., organic acids, alcohols), which can be directed to fuel production, materials precursors, or nutrient recycling (Detrell, 2021; Fahrion et al., 2021; Santomartino et al., 2023).

CO_2_ is also the principal abiotic carbon reservoir for ISRU, particularly on Mars, where it constitutes ~95% of the atmosphere, and can be harvested at relatively low energy cost compared with solid or trace volatile carbon sources (Owen et al., 1977; Hoffman et al., 2022). By contrast, this framing does not extend directly to the Moon, where the absence of a substantial atmosphere strongly limits the availability of accessible CO_2_ for fuel synthesis or other carbon-fed conversion pathways. Compressed CO_2_ can function as a working fluid for thermal systems and pressure control, having its main strategic role as feedstock for synthetic fuels and carbon-based materials, especially in Martian scenarios (Huang and Tan, 2014). For instance, methanation and Fischer–Tropsch type processes convert CO_2_ and CO into CH_4_ and hydrocarbon fractions when coupled to locally produced H_2_ (Holladay et al., 2007; Yao et al., 2010).

#### 3.2.2 Hydrogen

Several microbial and algal pathways are capable of H_2_ production and are considered a promising application at the interface with ISRU strategies (Khetkorn et al., 2017; Kosourov et al., 2021). Anaerobic hydrogen production via microbial fermentation (e.g., from the *Clostridia* genus) converts carbohydrates into H_2_, CO_2_, and organic acids (Romano et al., 2010; Ghimire et al., 2015). Light-driven H_2_ production by anoxygenic phototrophic bacteria, such as the *Rhodobacter* genus, can further convert acids derived from fermentation into additional H_2_ (Keskin et al., 2011). Green algae (i.e., *Chlamydomonas reinhardtii*) and cyanobacteria (e.g., *Synechocystis, Anabaena*, and *Nostoc* genera) can produce H_2_ through biophotolysis, where photosynthetic electron flow is redirected to [FeFe] or [NiFe]-hydrogenases under low O_2_ and sulfur concentration (Tamagnini et al., 2002; Carrieri et al., 2011; Skjånes et al., 2013; Cascone et al., 2025). H_2_ production mediated by nitrogenases during N_2_ fixation represents an alternative source despite the high ATP cost (Noar et al., 2015; Threatt and Rees, 2023). These pathways allow microbial biological systems to convert CO_2_ and reduced carbon intermediates derived from biomass or waste organics into H_2_ without high-temperature reactors (Jelen et al., 2016). Under optimized terrestrial dark fermentation conditions, continuous hydrogen production rates in mixed-culture systems reached 8.80 L H_2_ L^−1^ d^−1^ on arabinose and 8.09 L H_2_ L^−1^ d^−1^ on xylose, with microbial communities dominated by *Clostridium* spp. (Zagrodnik and Sobociński, 2024). In a separate optimized *Clostridium beijerinckii* process, cumulative H_2_ production increased from 1195 mL L^−1^ ± 45 mL L^−1^ to 5893 mL L^−1^ ± 25 mL L^−1^, with a yield of 2.16 mol H_2_ mol^−1^ glycerol (Khalil et al., 2023). These data indicate that biological H_2_ production is quantitatively feasible, but more plausibly relevant to supplementary, loop-coupled production than to primary mission-scale H_2_ supply.

H_2_ is central to abiotic ISRU because it supplies energy, reversible chemical energy storage opportunity, and reducing equivalents for chemical reactions (Zubrin et al., 2013; Wang et al., 2016). It could be obtained by electrolysis of H_2_O extracted from ice deposits, hydrated minerals, or recycled process water, linking H_2_ production to O_2_ generation infrastructure and to the recycling of H_2_O (Zubrin et al., 2013; Lomax et al., 2022). The feasibility of H_2_-based ISRU is strongly constrained by local H_2_O accessibility and by the energy costs of extraction, purification, and electrolysis. In reduced-gravity electrolysis experiments relevant to lunar and Martian applications, performance under lunar gravity decreased by ~11% when the additional overpotential was not compensated, whereas maintaining the same current density required only ~1% additional power, indicating that reduced gravity itself may be a secondary constraint relative to water access and system energy demand (Lomax et al., 2022).

H_2_ might also be sourced from geological reservoirs. On Earth, diverse processes produce H_2_ in large quantities, including water-rock reactions analogous to serpentinization, radiolysis of water, and mechanoradical generation (Wong et al., 2025). These processes may be present on other planetary bodies and could provide energy (Vance et al., 2016; Tarnas et al., 2018). Although controlled exploitation of such processes *in situ* under space conditions remains speculative, geological hydrogen utilization in ISRU scenarios should be regarded as prospective rather than operational (Cannat et al., 2010; Klein et al., 2013; Giovannelli et al., 2025). Once available, H_2_ can be used to feed Sabatier and Fischer–Tropsch reactors, support metallurgical and electrolytic reduction of regolith-derived oxides, and be recombined with O_2_ in fuel cells (Yao et al., 2010; Zubrin et al., 2013; Wang et al., 2016; Sargeant et al., 2021; Ullah et al., 2025). The dominant engineering challenges are associated with storage and distribution (Sakintuna et al., 2007; Li et al., 2025), while geological challenges are related to the identification and exploitation of geological reservoirs (Everts et al., 2025).

#### 3.2.3 Methane

CH_4_ functions both as an ascent/transfer propellant and as a medium-term energy buffer for intermittent power sources (Alarcón et al., 2020; Alam et al., 2022). Methanogenic archaea provide a direct biotic pathway from CO_2_ and simple organics to CH_4_ (Thauer et al., 2008; Lira-Silva et al., 2012; Feregrino-Mondragón et al., 2023). Hydrogenotrophic methanogens (e.g., *Methanobacterium* and *Methanococcus* genera) reduce CO_2_ with H_2_, while acetoclastic and methylotrophic methanogens (i.e., *Methanosarcina* genus) convert acetate or methylated compounds into CH_4_ and CO_2_ (Thauer et al., 2008). In bioengineered systems, these microbial metabolisms are implemented in anaerobic digesters that process mixed organic waste streams into a CH_4_ and CO_2_-rich biogas (Batstone et al., 2002; Chynoweth et al., 2006). Reported anaerobic-digestion methane yields, generally in the range of 467 mL−680 mL CH_4_ g^−1^ volatile solids, support the relevance of biomethane for waste stabilization and supplementary energy recovery, but do not support its interpretation as a primary propellant source in space (Browne and Murphy, 2013; Zhang et al., 2019). For space missions, such reactors are more plausibly relevant to waste stabilization and supplementary energy production than to mission-scale propellant generation, because CH_4_ output from internal loop-closure streams is intrinsically constrained by the limited carbon inventory of crew metabolism and inedible biomass. Thus, biologically produced CH_4_ is better viewed as a low-throughput, loop-coupled energy carrier or fuel-cell feedstock than as a primary propellant source (Chynoweth et al., 2006; Menezes et al., 2015). Methane is an end-product in many ISRU operations. It is compatible with liquid oxygen (LOX)/CH_4_ propulsion and offers higher volumetric energy density. (Moore, 2010; Pizzarelli and Battista, 2023). From the stoichiometry of Sabatier methanation, 1 mol CO_2_ and 4 mol H_2_ are required to produce 1 mol CH_4_, corresponding to 44 g CO_2_ and 8 g H_2_ per 16 g CH_4_. A MAV-class ascent propellant load requires approximately 7.0 t CH_4_, corresponding to ~19.3 t CO_2_ and ~5.25 t C (Kleinhenz and Paz, 2017). If lunar regolith contains carbon at ~100 μg g^−1^-200 μg g^−1^, consistent with Apollo soil values averaging ~124 ppm C ± 45 ppm C and broader lunar soil/regolith-breccia values reaching up to ~280 ppm C, this carbon demand would require processing ~2.6 × 10^4 − 5^ t 0.3 × 10^4^ t of regolith even under ideal complete recovery (Gibson and Chang, 1992; Thomas-Keprta et al., 2014). Non-ideal extraction and conversion efficiencies would push the required processed mass toward 10^5^ t−10^6^ t. This requirement makes CH_4_ production strongly dependent on carbon and H_2_O availability, and therefore highly location-specific. In Mars ascent concepts, CH_4_ is produced primarily via Sabatier methanation of captured CO_2_ with electrolytic H_2_, coupled with the co-produced H_2_O recycled to electrolysis (Alam et al., 2022; Ullah et al., 2025). This framing is most credible for Mars, where atmospheric CO_2_ is abundant. On the Moon, the carbon mass balance makes propellant-scale CH_4_ production unlikely without substantial external carbon input. Fischer–Tropsch synthesis from CO/CO_2_-derived syngas provides access to more complex hydrocarbons (Yao et al., 2011; Kaiser et al., 2013). For the same reason, Fischer–Tropsch pathways are also far more aligned with Martian than lunar resource scenarios. The strong exothermicity of CO_2_ methanation makes heat management and catalyst stability central drivers for reactor design (Alarcón et al., 2020; Huynh and Yu, 2020).

### 3.3 . Materials

Structural and functional materials (e.g., metals, ceramics, polymers, and composites) constitute the backbone of all engineering systems, habitat construction, radiation shielding, mechanical components, and energy storage assemblies (Ferrone et al., 2022; Stapperfend et al., 2023). The conventional Earth-dependent supply chain could ideally be replaced by a self-sufficient system, in which collection, refinement, and production of materials are available *in situ*. Among the abiotic ISRU systems proposed, the Regolith Advanced Surface Systems Operations Robot (RASSOR) and the ISRU Pilot Excavator (IPEx) represent an approach to autonomous excavation and material handling in low-gravity environments (Mueller et al., 2013).

Biological systems enable the production of a broad spectrum of materials that extend beyond elemental extraction and support structural, functional, and manufacturing needs in space missions (Brandl et al., 2001; Arif et al., 2023; Rendón-Castrillón et al., 2023). Microorganisms can convert inorganic substrates and waste streams into polymers, mineral phases, and composite materials under a wide range of temperature and pressure (Poirier et al., 1995; Cheng et al., 2013; Di Donato et al., 2014; Finore et al., 2015; Li et al., 2016). These microbial-produced materials complement conventional production, reducing thermal, power, and hardware requirements.

#### 3.3.1 Biomining

**Figure 2:**
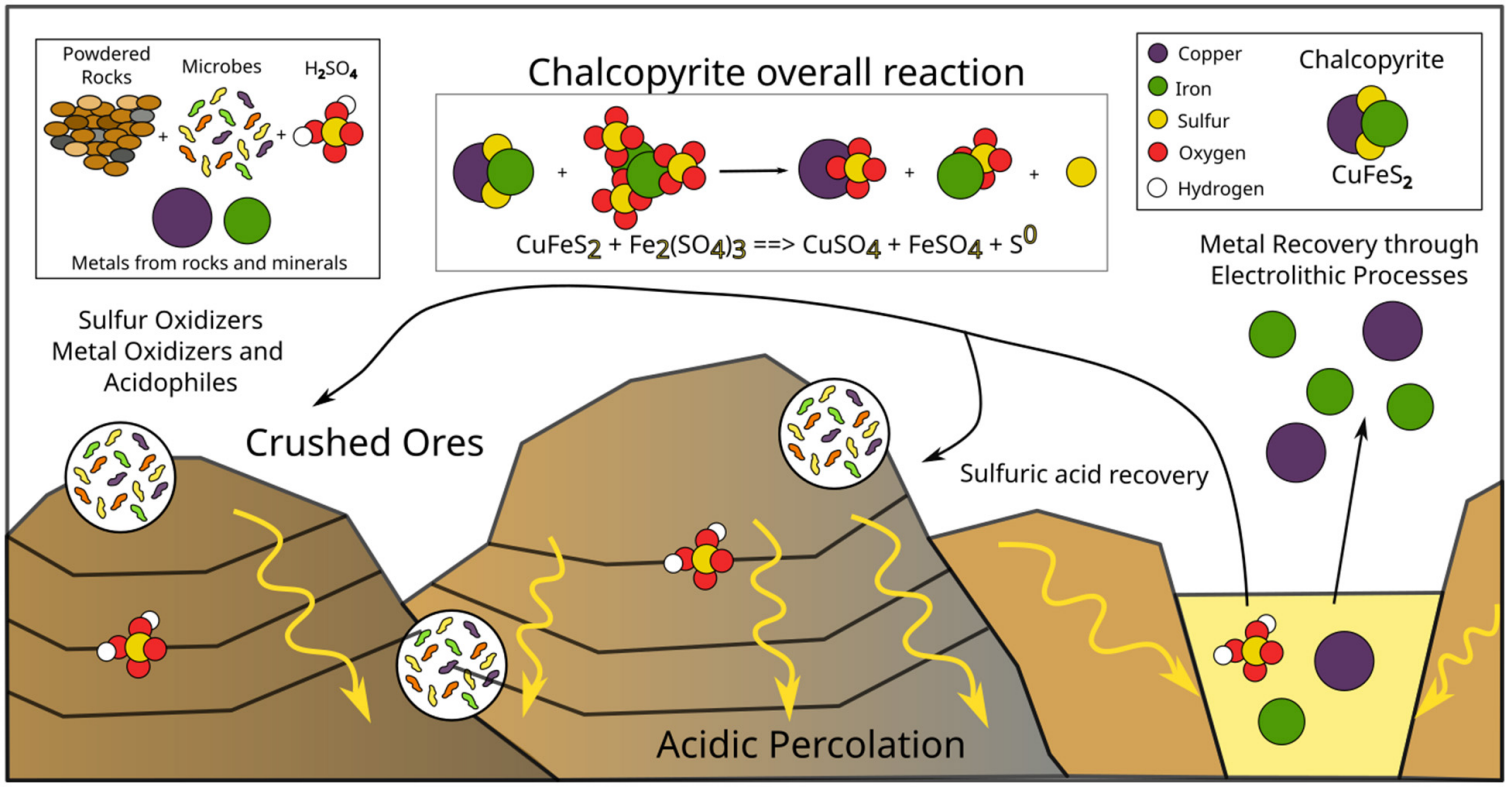
General chemolithotrophic chalcopyrite bioleaching mechanism. Microbes oxidize sulfide minerals present in crushed rocky ores, producing H_2_SO_4_ and Fe^3+^, which facilitate dissolution of CuFeS_2_ and release of Cu ions. The image shows microbial colonization, acidic percolation, and metal solubilization stages. The crystal structure of CuFeS_2_ has been simplified for illustration purposes; it has a tetragonal crystal lattice structure.

Microbial-based biotechnologies have demonstrated their high efficacy in several applications and may become a key component of future ISRU strategies (Anand et al., 2012; Cockell et al., 2021; Kaksonen et al., 2021; Roberto and Schippers, 2022; Guerrero-Gonzalez and Zabel, 2023; Santomartino et al., 2023). Minerals and metals are fundamental in construction, infrastructure development, and additive manufacturing (AF), e.g., 3D metal sintering (Ngo et al., 2018; Sacco and Moon, 2019; Hedayati and Stulova, 2023; Tonietti et al., 2024, 2026). Biomining (Figure 2) refers to the utilization of microorganisms to extract and recover valuable metals from minerals and waste materials (Bosecker, 1997; Johnson, 2010, 2014; Cockell et al., 2020; Rendón-Castrillón et al., 2023; Tonietti et al., 2024). On Earth, around 20 %−25 % of global Cu and 5 % of Au are extracted using this technique (Johnson, 2010). This process has also been applied to the leaching of Fe, Cu, Ni, Co, Zn, and U, and for the pre-treatment of challenging sulfidic Au ores (Gan et al., 2015; Srichandan et al., 2019; Cockell et al., 2021; Tonietti et al., 2024). Biomining can provide economic and environmental advantages compared to other metal extraction methods, e.g., pyrometallurgy (Srichandan et al., 2019). In addition, it can be used as a complementary technique to extract trace metals from mine waste, low-grade ores, and from e-waste (i.e., electronic waste), which has emerged as one of the main categories of solid waste in recent decades (Bosecker, 1997; Brandl et al., 2001; Srichandan et al., 2019; Roberto and Schippers, 2022).

If applied to space, biomining could be harnessed for ISRU, offering an alternative approach for selectively extracting resources in space or producing high-quality metal concentrates (Gumulya et al., 2022; Santomartino et al., 2022, 2026). On board the ISS, experiments have demonstrated the principle of biological mining of metals such as V, platinum group elements and Rare Earth Elements (REEs) from basaltic and asteroidal substrates (Cockell et al., 2020, 2021; Santomartino et al., 2024, 2026). In the BioRock experiment, *Sphingomonas desiccabilis* enhanced REE release from basalt to 111.9%−429.2% of non-biological controls, although the absolute extracted fraction remained low, at approximately 0.024%−0.117% of the total REE inventory (Cockell et al., 2020). Vanadium leaching was also enhanced by *S. desiccabilis* and *Bacillus subtilis*, reaching 184.92%−283.22% of abiotic controls (Cockell et al., 2021). Additional terrestrial bioleaching experiments using eucrite and impact-crater analogs showed that *S. desiccabilis* extracted up to ~10.6% of Ce and ~1.5% of Th from different substrates (Tonietti et al., 2026). More recently, fungal biomining of L-chondrite material on the ISS showed that *Penicillium simplicissimum* could extract 19.29% Ru, 11.91% Pd and 0.29% Pt from the meteorite under microgravity (Santomartino et al., 2026). These values indicate proof-of-concept, providing quantitative evidence that microbial leaching can enhance metal mobilization from planetary analog and extraterrestrial substrates under spaceflight-relevant conditions.

Biomining can have lower thermal and chemical demands than pyrometallurgical routes, which could be an advantage in space, where energy, reagents, and engineering capacity are constrained. The most used and characterized biomining organisms are autotrophic iron-oxidizing and sulfur-oxidizing microorganisms, which thrive at low pH. These pH values allow metallic ions to remain in solution, although neutral pH biomining with heterotrophic species is also possible and may expand the range of elements that can be extracted in environments characterized by heterogeneous mineral compositions (Cockell et al., 2020; Santomartino et al., 2020). Representative biomining microorganisms include the mesophilic bacteria *Leptospirillum ferrooxidans*, the acidophilic bacteria *Acidithiobacillus ferrooxidans*, and the thermophilic chemolithotroph *Acidianus brierleyi* (Johnson, 2010, 2014; Vilhelmsson et al., 2023). Unlike chemical-intensive extraction processes, bioleaching can produce fewer toxic byproducts, reduce greenhouse gas emissions, and mitigate the environmental impact of mining by limiting the need for high-temperature smelting or hazardous chemical leaching (Rendón-Castrillón et al., 2023; Wu et al., 2025).

#### 3.3.2 Space biomanufacturing and biopolymers

Space biomanufacturing can be defined as the use of biological systems to convert *in situ* resources and recycled mission waste into mission-relevant products, extending biology beyond extraction and waste treatment to the *de novo* synthesis of materials, food, and therapeutics under the mass, power, volume, and LC constraints of long-duration exploration architectures (Nangle et al., 2020; Berliner et al., 2021, 2022; Averesch et al., 2023). Its relevance spans both lunar and Martian missions, although the suitability of individual pathways is location-dependent. Mars represents the more direct target for carbon-fed biomanufacturing because atmospheric CO_2_ can be coupled to microbial conversion, whereas lunar implementations will likely depend more heavily on recycled carbon streams and closer integration with upstream ISRU and habitat-recycling modules (Averesch, 2021; Berliner et al., 2021, 2022; Averesch et al., 2023).

Microbial selection is a central design variable in space biomanufacturing. Organism choice should not default to conventional industrial models. Instead, it should be guided by metabolic compatibility with ISRU and LC-derived feedstocks, as well as by process scale, continuity, and scenario-specific environmental constraints (Averesch, 2021; Averesch et al., 2023). Although model organisms such as *Escherichia coli* and *Pichia pastoris* can be engineered for CO_2_-based growth, naturally adapted carbon-fixing or mixotrophic organisms may offer more direct advantages where single-carbon feedstocks dominate, and engineering overhead must be minimized (Gleizer et al., 2019; Gassler et al., 2020; Pavan et al., 2022). This logic is especially relevant for Mars-oriented bioproduction, where cyanobacterial platforms have been proposed and experimentally evaluated under Mars-relevant resource conditions (Ramalho et al., 2022a,b). In parallel, metabolic engineering may expand the usable carbon space of biomanufacturing systems by enabling the upcycling of non-natural feedstocks, including synthetic plastics (Sullivan et al., 2022; Tiso et al., 2022).

Biopolymers are particularly relevant because they can be produced through biological conversion of recycled or *in situ* feedstocks. Among microbial biopolymers, polyhydroxyalkanoates (PHAs) and bacterial cellulose represent complementary material platforms. PHAs are biodegradable polyesters accumulated intracellularly by diverse microorganisms. Bacterial cellulose, instead, is an extracellular nanofibrous polysaccharide, valued for its high H_2_O retention, mechanical robustness, and potential use in biomedical and functional materials (Li et al., 2016; Rosenboom et al., 2022; Yan et al., 2026). The strategic importance of these materials for space missions is reinforced by mission-level analyses showing that plastics account for an increasingly large fraction of the logistics burden, rising from approximately 19% to 37% of total mass in longer-duration lunar scenarios and from 48% to 60% in Martian scenarios. These estimates support prioritizing polymer-focused biomanufacturing for thermoplastic feedstocks such as PHAs, which could supply high-turnover items and replacement parts via additive manufacturing (Averesch et al., 2023).

At the process level, a Mars-oriented two-module route has already demonstrated coupling between carbon capture and biopolymer synthesis, with *Sporomusa ovata* producing acetate from CO_2_ at 10.4 mmol L^−1^ d^−1^ and the resulting unprocessed medium supporting PHB production by *Cupriavidus basilensis* at 12.54 mg L^−1^ h^−1^, with an overall carbon yield of 11.06% from acetate. The same study further estimated that eliminating intermediate purification and filtration steps could reduce process mass by up to 98% (Cestellos-Blanco et al., 2021). Bacterial cellulose provides a complementary option because its mechanical integrity can be retained under space-relevant stressors. After long-term spaceflight and Mars-like exposure, cellulose integrity and mechanical properties were not significantly altered. However, *de novo* cellulose production was reduced by a factor of 1.5, and dry cellulose yield from reisolated *Komagataeibacter oboediens* was reduced by a factor of 1.7 relative to the wild type, indicating that the material itself remains promising, although productivity is sensitive to extraterrestrial conditions (Orlovska et al., 2021).

Experimental evidence already supports Mars-relevant production routes and shows that these materials remain stable under space and Mars-like conditions. Routine deployment on the Moon or Mars will require further progress in scale-up, long-term process stability, autonomy, and systems integration (Averesch, 2021; Cestellos-Blanco et al., 2021; Berliner et al., 2022; Averesch et al., 2023).

#### 3.3.3 Regolith advanced surface systems operations robot (RASSOR)

RASSOR is a robotic excavation system developed for operation on the Moon and Mars (Mueller et al., 2013, 2017; Radl et al., 2023). RASSOR is essential to processes that extract resources such as O_2_, H_2_O, and construction materials from planetary surfaces. Designed for operation in low-gravity environments, it utilizes a counter-rotating bucket drum system that provides both excavation and traction for efficient material collection without requiring heavy downward force (Mueller et al., 2013). The system can be coupled with ISRU processing plants, delivering raw regolith for further refinement into breathable O_2_, rocket fuel, or habitat construction materials (Mueller et al., 2013; Chambers et al., 2018; Nasr et al., 2018; Wang et al., 2022). Due to its lightweight design, RASSOR can only carry a relatively small amount of regolith, requiring multiple excavation cycles to meet resource processing demands. Nevertheless, Martian dust is highly abrasive and electrostatically charged, potentially being hazardous for RASSOR's moving parts, sensors, and electronics (Méndez Harper et al., 2021; Suman and Zanini, 2024).

#### 3.3.4 ISRU pilot excavator (IPEx)

NASA's ISRU Pilot Excavator (IPEx) is a prototype robotic excavation system developed to advance the TRL of regolith acquisition and handling for ISRU on the lunar and martian surface (Schuler et al., 2024). Conceived as a technology pathfinder, IPEx is intended to characterize excavation dynamics, tool-soil interactions, and the mechanical robustness of excavation strategies under simulated low-gravity and vacuum conditions (Mueller, 2023; Schuler et al., 2024). Its modular and scalable architecture facilitates refinement of excavation tools, control algorithms, and system-level integration within broader ISRU architectures. As a pilot-scale platform, IPEx remains constrained in excavation volume, continuous operational duration, and autonomous capability relative to prospective production-scale systems (Cloud et al., 2025).

#### 3.3.5 PROSPECT (package for resource observation and in-situ prospecting for exploration, commercial exploitation and transportation)

PROSPECT is an ISRU technology demonstrator, led by ESA, designed to investigate and characterize lunar regolith with a specific focus on subsurface resource prospecting and sample processing in support of surface operations (Carpenter and Fisackerly, 2017; Trautner et al., 2024). The system integrates a drilling assembly, known as the ProSEED drill, with a sample processing and analysis laboratory (ProSPA), designed for the acquisition of regolith samples from depths of up to approximately 1 m (Ricardo et al., 2023). These samples are then transferred to an onboard processing unit, where thermal extraction methods are used to release volatiles and analyze their composition (Trautner et al., 2024). A key strength of PROSPECT lies in its ability to perform controlled subsurface sampling and *in situ* regolith processing while maintaining high analytical fidelity, enabling detailed assessment of regolith stratigraphy, mechanical behavior, and resource potential (Heather et al., 2021; Trautner et al., 2024).

#### 3.3.6 Other technologies

Among the currently explored approaches, two further techniques are representative of the diversity in excavation and sampling strategies: (i) TRIDENT (The Regolith and Ice Drill for Exploring New Terrain), and (ii) PlanetVac, developed by HoneyBee Robotics. TRIDENT, developed for NASA's VIPER rover, is a rotary-percussive drill capable of sampling down to 1 m depth while maintaining stratigraphic integrity in icy regolith environments (Paulsen et al., 2018; Zacny et al., 2022). PlanetVac is a gas-driven pneumatic sampling system able to collect loose regolith (Zacny et al., 2024).

### 3.4 Food

Long-duration human spaceflight demands food systems that extend beyond storage and resupply, requiring integrated strategies for preservation, nutrient retention, and *in situ* production (Douglas et al., 2021; Ellena et al., 2024; Zhao, 2024). Although food production is not an ISRU function *per se*, biological food systems will likely need to interface with ISRU resource flows to obtain nutrients, H_2_O, and gases, thus constituting a critical component of coupled architectures. Conventional approaches based on pre-packaged terrestrial supplies are constrained by mass, volume, degradation over time, and nutrient loss, becoming increasingly impractical for extended missions and planetary habitation (Cooper et al., 2017; Carillo et al., 2020). Closed-loop food production and preservation systems can mitigate these constraints by supporting nutrient retention and regenerating edible biomass from resources circulating within the habitat (Vermeulen et al., 2023; Ellena et al., 2024; Frossard et al., 2024). Beyond sustaining caloric intake, fresh food production can contribute positively to crew morale, cognitive performance, and mental health by alleviating sensory monotony in isolated and confined environments (Landon et al., 2025). Food production in coupled ISRU-BLSS architectures should include both microbial phototrophic platforms and higher-plant systems, because they address different nutritional and operational functions. Microalgae and cyanobacteria-based photobioreactors can support rapid biomass generation, nutraceutical production, and nutrient recovery from recycled waste streams (Detrell, 2021; Fahrion et al., 2021; Sachdeva et al., 2021; Revellame et al., 2024; Ellena et al., 2025). Crop systems such as VEGGIE, APH, and LADA remain essential for fresh vegetables, dietary variety, sensory quality, and psychological support (Massa et al., 2020; De Micco et al., 2023; Landon et al., 2025).

#### 3.4.1 Microalgae and cyanobacteria

Microalgae and cyanobacteria provide a compact food-production route for BLSS and coupled ISRU-BLSS architectures, especially where biomass generation, nutrient recovery, and reactor control must be integrated within limited volume (Detrell, 2021; Fahrion et al., 2021; Revellame et al., 2024). Their main advantage is rapid biomass turnover, high edible fraction, compatibility with closed photobioreactor operation, and the possibility of coupling food production with CO_2_ removal, O_2_ generation, and nutrient recovery (Fahrion et al., 2021; Revellame et al., 2024). This biomass can supply proteins, essential amino acids, vitamin-related compounds, carotenoids, phycobiliproteins, phenolic compounds, tocopherols, and polyunsaturated fatty acids (Villarruel-López et al., 2017; Hachicha et al., 2022; Revellame et al., 2024; Ellena et al., 2025). For *Chlorella*-based lunar life-support concepts, microalgal biomass has been estimated to substitute up to approximately 35% of daily food mass, while a photobioreactor sized to provide 10%−30% of daily food consumption would require approximately 50 L−100 L of culture volume per person (Detrell, 2021). Mission mass analyses further indicate that photobioreactors become advantageous mainly in long-duration scenarios, with reduced food resupply estimated to offset the added system mass after approximately 4.0 years−6.5 years when providing 30% of the dietary supply and 4.8 years−7.3 years when providing 10% (Detrell, 2021). Mars-oriented modeling further emphasizes that cyanobacterial production must be treated as a constrained resource-conversion process, with performance depending on H_2_O, light, temperature, regolith-derived minerals, perchlorates, and atmospheric carbon and nitrogen availability (Ramalho et al., 2024).

Their relevance also derives from their ability to convert simple external or recycled resource streams into edible or processable biomass. Cyanobacteria have been proposed as primary converters for Mars because selected strains could use atmospheric CO_2_, potentially supplemented nitrogen sources, locally recovered H_2_O, and inorganic nutrients derived from regolith, thereby linking external resource acquisition to internal biological production (Verseux et al., 2016, 2024). This rationale is further supported by space-exposure and Mars-simulation studies showing that selected phototrophs and extremotolerant cyanobacteria, including *Chroococcidiopsis*, can retain viability or biosignature-relevant integrity after exposure to low Earth orbit or Mars-like conditions (Cockell et al., 2011; de Vera et al., 2019). *Anabaena* sp. PCC 7938 grew under a low-pressure N_2_/CO_2_ atmosphere compatible with Mars-oriented photobioreactor concepts and used MGS-1 Mars regolith simulant as a nutrient source (Verseux et al., 2021). *Chroococcidiopsis* sp. 029 was cultivated with lunar and Martian regolith simulants supplemented with synthetic human urine as a nitrogen source, and the resulting cyanobacterial biomass supported secondary bacterial production (Fernandez et al., 2023). *Synechococcus nidulans* was cultivated in a simulated Martian medium prepared from regolith leachate and astronaut urine, supporting the feasibility of reducing Earth-supplied nutrients through local mineral inputs and recycled waste streams (Concas et al., 2023). In a related Mars-relevant context, perchlorate-tolerant *Chroococcidiopsis* sp. CCMEE 029 accumulated sucrose after air drying, and the resulting lysate supported bacterial growth, suggesting that extremotolerant cyanobacteria could provide substrates for heterotrophic BLSS components under chemically stressful Martian conditions (Billi et al., 2021). Similar cultivation concepts have been evaluated for *Chroococcidiopsis thermalis* CCALA 050 using Martian regolith simulant leachate and synthetic human urine, with potential implications for food and O_2_ production during Mars missions (Fais et al., 2024). In MELiSSA-related work, *Limnospira indica* PCC8005 was tested for 35 days in a simplified ground demonstrator using recycled CO_2_ and urine-derived nitrogen sources, supporting its role in air revitalization, edible biomass production, and recovery of waste-derived nitrogen (Poughon et al., 2021; Sachdeva et al., 2021; Ellena et al., 2024).

Compared with higher plants, phototrophic microbial systems offer greater volumetric compactness, simpler modularization, continuous or semi-continuous harvesting, and tighter control of gas-liquid exchange (Detrell, 2021; Fahrion et al., 2021). This contrasts with crop systems, where edible dry biomass production in preliminary VEGGIE testing ranged from approximately 3.5 g m^−2^ d^−1^-8 g m^−2^ d^−1^ depending on species, reflecting the larger illuminated areas and longer developmental cycles required by higher plants (Massa et al., 2016). Photobioreactors do not replace crop systems, because higher plants provide fresh vegetables, fiber, dietary structure, sensory variety, and psychological benefits that microbial biomass alone is unlikely to reproduce fully (Massa et al., 2020; Douglas et al., 2021; Landon et al., 2025). Photobioreactors also introduce specific constraints related to light attenuation in dense cultures, mixing energy, gas-liquid mass transfer, contamination control, biomass harvesting, dewatering, palatability, and long-term culture stability (Detrell, 2021; Fahrion et al., 2021). Reduced-gravity effects may further constrain performance, as simulated microgravity experiments with *Limnospira indica* showed slower growth under low-shear conditions, probably linked to gas-exchange limitations and O_2_ accumulation (Ellena et al., 2024). Their technological maturity also differs, with VEGGIE, APH, and LADA already operated on the ISS, and most food-oriented microalgal or cyanobacterial photobioreactor concepts still relying on ground demonstrations, analog tests, or partial BLSS studies (Levinskikh et al., 2005; Massa et al., 2020; Bunchek et al., 2021; Sachdeva et al., 2021; Ellena et al., 2024).

#### 3.4.2 Vegetable production system (VEGGIE)

VEGGIE is a bioregenerative life support-relevant system developed by NASA for *in situ* crop cultivation in microgravity and planetary environments (Massa et al., 2016; Ehrlich et al., 2017; Bunchek et al., 2021). This system is a core BLSS component that could interface with ISRU-derived resource streams (e.g., CO_2_ and H_2_O) by providing O_2_, fresh food, and psychological benefits for crew members. VEGGIE is designed as a modular plant growth chamber that operates under controlled environmental conditions, utilizing LED lighting, regulated nutrient delivery, and optimized atmospheric composition to facilitate plant growth in space (Massa et al., 2016; Zabel et al., 2016). The VEGGIE chamber consists of a growth unit with an open architecture, allowing plants to interact with the surrounding cabin environment. A root mat irrigation system ensures adequate H_2_O and nutrient delivery in microgravity. This approach enables plant roots to anchor effectively and absorb nutrients despite the absence of gravity-driven fluid dynamics that usually impact plant growth (Massa et al., 2016, 2020). VEGGIE contributes to bioregenerative air revitalization, as plants consume CO_2_ and generate O_2_, directly contributing to the atmospheric balance of spacecraft (Johnson et al., 2021; Kordyum and Hasenstein, 2021). Beyond its space applications, VEGGIE technology has significant terrestrial benefits, particularly in controlled environment agriculture, urban farming initiatives, and in harsh environments (e.g., deserts and polar regions) (Zabel et al., 2016).

#### 3.4.3 Advanced plant habitat (APH)

The Advanced Plant Habitat (APH) is a comprehensive plant-growth facility deployed in LEO. The APH facility is a growth chamber that can host plants under variable environmental controls (i.e., spectral quality, light intensity, temperature, relative humidity, CO_2_, and C_2_H_4_ concentration) for life cycles as long as 135 days (Morrow et al., 2016; Monje et al., 2020). APH houses a 0.2 m^2^ plant canopy and replaceable Science Carriers preloaded with porous clay substrate and fertilizer. It employs a multi-spectral LED array to optimize photosynthesis and morphogenesis under remote control (Morrow et al., 2016; Monje et al., 2020). The system's “Water Recovery and Distribution Subassembly” (WRDS) uses peristaltic pumps and capillary tubing to deliver nutrient solutions, while the Air Filtration Assembly (AFA) and CO_2_ regulation modules maintain atmospheric balance (Johnson, 2013; Monje et al., 2019, 2020). APH has demonstrated complete life-cycle cultivation of both model and crop species, *Arabidopsis thaliana* and *Triticum aestivum*, showing that germination can occur entirely in microgravity under fully automated planting and watering protocols (Morrow et al., 2016; Monje et al., 2019, 2020).

#### 3.4.4 LADA greenhouse

The LADA greenhouse is a Russian bioregenerative plant cultivation system designed to support *in situ* food production and atmospheric revitalization under microgravity conditions aboard the ISS (Bingham et al., 2002, 2003). The system focuses on evaluating the physiological, morphological, and biochemical responses of plants to reduced gravity, aiming to study the feasibility of sustainable crop production for long-duration missions and planetary habitation scenarios (Levinskikh et al., 2005; Sychev et al., 2007). LADA operates as a closed, regulated growth chamber that integrates controlled atmospheric composition, T regulation, humidity control, and optimized illumination to create a stable environment for plant development (Bingham et al., 2002, 2003). The system utilizes a root module containing capillary nutrient delivery substrates, which ensures efficient H_2_O and nutrient transport in the absence of buoyancy (Berkovich et al., 2015). Although limited in scale and crop throughput, LADA has demonstrated high operational reliability and longevity to be considered a precursor system for a more advanced lunar and Martian surface greenhouse.

### 3.5 Energy

*In situ* energy production is fundamental for sustainable extraterrestrial operations and can rely on both abiotic and biotic conversion pathways. Solar power introduces significant constraints, as illumination cycles, dust deposition, and latitude-dependent insolation can limit energy availability. Processes such as serpentinization can yield H_2_, which serves as both a clean energy source and a key reactant for metallurgical reduction and chemical synthesis (Wong et al., 2025). Electrolysis of locally sourced water ice provides another direct pathway to H_2_ and O_2_, supporting both fuel cell operation and propellant production. The combination of H_2_ with carbon-bearing species through Fischer–Tropsch synthesis enables the generation of CH_4_ and hydrocarbons. In parallel, microbial and photosynthetic systems can generate energy-dense biofuels from CO_2_, H_2_ or organic substrates, producing CH_4_, alcohols or lipid-derived hydrocarbons. Nuclear fission reactors, radioisotope thermoelectric generators, and thermal energy storage systems can provide baseline or backup power independent of environmental conditions (Oleson et al., 2022; El-Genk and Schriener, 2024).

#### 3.5.1 Biofuels

Biofuels comprise a broad class of energy-rich compounds derived from biological substrates, including alcohols (e.g., bioethanol, biobutanol), fatty-acid esters (biodiesel), gaseous products such as CH_4_ and H_2_, and hydrocarbons that mimic conventional fossil-derived fuels (Neupane, 2022; El-Araby, 2024). Biofuel production relies on biochemical or thermochemical conversion pathways that transform organic matter into combustible molecules (Ma and Hanna, 1999; El-Araby, 2024). These pathways include microbial fermentation of sugars to alcohols, transesterification of lipid-rich biomass into fatty-acid methyl esters, anaerobic digestion of organic waste into methane-rich biogas, and photosynthetically driven synthesis of reduced carbon compounds in algae or cyanobacteria (Ma and Hanna, 1999; Brennan and Owende, 2010; Negri et al., 2020; Finore et al., 2023). Recent studies have expanded these processes toward metabolic pathways optimized for high yield, light utilization, or tolerance to extreme conditions (Rabinovitch-Deere et al., 2013; Das et al., 2020; Meng, 2023). On Earth, biofuel production is well established in industrial infrastructures to supply energy for transportation, heating, and electricity generation (Cadenas and Cabezudo, 1998; El-Araby, 2024). The theoretical maximum ethanol yield from hexose sugars is 0.51 g ethanol g^−1^ sugar, providing a useful upper bound for fermentative alcohol production from carbohydrate-rich feedstocks (Gombert and van Maris, 2015). Fermentative production of C_2_H_5_OH from corn and sugarcane constitutes the largest global biofuel sector, while biodiesel derived from vegetable oils or waste fats provides a renewable alternative to petroleum diesel (Ma and Hanna, 1999; Neupane, 2022; El-Araby, 2024). Anaerobic digesters convert agricultural waste, sewage sludge, and food residues into biogas, which can be combusted for heat and power or refined into biomethane for grid injection and vehicle propulsion (Negri et al., 2020; Begum et al., 2024). In food-waste systems, reported biomethane yields are commonly on the order of 268 mL CH_4_ g^−1^ ± 199 mL CH_4_ g^−1^ to 480 mL CH_4_ g^−1^ ± 88 mL CH_4_ g^−1^ VS, increasing on average to 406 mL CH_4_ g^−1^ ± 137 mL CH_4_ g^−1^ VS under co-digestion, which is consistent with a role in waste valorization and supplementary energy recovery rather than large-scale fuel supply (Negri et al., 2020). Algae-based hydrocarbon synthesis or microbial consortia could minimize resource use and substantially reduce carbon emissions (Greenwell et al., 2009; Brennan and Owende, 2010; Das et al., 2020; Wang et al., 2024). Microbial methanogenesis can convert CO_2_ and H_2_ into CH_4_, producing a viable propellant for vehicles (Lecker et al., 2017; Jensen et al., 2021; Koehle et al., 2023). However, in space systems, this route is most credible where carbon and hydrogen feedstocks are externally available at a sufficient scale, especially in Martian scenarios that provide abundant atmospheric CO_2_. In contrast, when biological fuels are derived primarily from internal loop-closure streams, their production is limited, and their most realistic role is therefore likely to be supplementary power generation, low-duty-cycle energy buffering, or feedstock recycling rather than propulsion (Koehle et al., 2023; Negri et al., 2020). Anaerobic digestion of crew-generated organic waste (e.g., inedible plant biomass, food scraps, fecal matter, and habitat effluents) can produce biogas mixtures that supplement energy generation or serve as feedstock (Negri et al., 2020; Koehle et al., 2023). In coupled BLSS-ISRU architectures, biological fuel production is scientifically credible as a loop-closing and waste-valorizing strategy, but its use as a mission-relevant propellant source remains limited or speculative unless supported by substantial external carbon and hydrogen inputs. Biological fuel production represents a means of closing metabolic loops, valorizing waste streams, and sustaining coupled BLSS-ISRU architectures.

#### 3.5.2 Fission surface power (FSP)

The FSP initiative addresses the availability of continuous, high-duty-cycle electrical power that is decoupled from local illumination, latitude, and dust-driven intermittency (Mason et al., 2008; Oleson et al., 2022). In the current NASA-DOE (Department of Energy) program, the objective is the design, fabrication, and ground testing of a ~40 kW_e_ class surface fission power system intended for a lunar demonstration in the early 2030s, with extensibility to Mars surface applications. FSP is framed as a generator enabling steady-state operation of energy-intensive unit operations while reducing operational complexity associated with energy storage. NASA has reported parallel investments in non-nuclear subsystems (e.g., power conversion and power management/distribution technologies) such as Brayton-cycle converters.

#### 3.5.3 UltraFlex

The UltraFlex solar arrays represent a class of flexible photovoltaic power systems that reduce the mass and volume typically associated with large-area solar energy collection (Spence et al., 2005; Franklin et al., 2006). UltraFlex implements a tensioned umbrella-shaped membrane formed by interconnected triangular blanket segments, which enables compact packaging and deployment into a relatively stiff, large-area structure (Spence et al., 2005, 2006). In next-generation UltraFlex-175 development studies, this architecture was reported to provide specific powers of approximately 175 W kg^−1^-220 W kg^−1^ at deployment, stowed power densities >33 kW m^−3^, and scalability beyond 7 kW wing sizes (Spence et al., 2005; White et al., 2007).

UltraFlex is now also established in flight applications. For example, the Phoenix Mars Lander demonstrated UltraFlex arrays with a reported power-to-mass ratio of approximately 100 W kg^−1^, and Northrop Grumman's Cygnus spacecraft employs two UltraFlex arrays producing approximately 3.5 kW of spacecraft power. These flight applications reinforce its relevance as a near-term, high-TRL solar option where nuclear baseload is unavailable (Oeftering et al., 2012; McNatt et al., 2016). For surface ISRU, shadowed regions, or dust-attenuated periods, UltraFlex remains constrained by the intrinsic intermittency of solar input and by site-specific illumination conditions, requiring energy storage or complementary baseload power for continuous operations (Spence et al., 2005; McNatt et al., 2016).

#### 3.5.4 Sabatier reaction

The Sabatier reaction (R8) converts electrically produced H_2_ and CO_2_ into CH_4_, which is a storable energy carrier compatible with long-duration storage and downstream reconversion to electricity (e.g., via combustion or fuel cells) (Ghaib et al., 2016; Rönsch et al., 2016).

CO_2_+4H_2_ → CH_4_+2H_2_O (R8)

Thermodynamically, CO_2_ methanation is strongly exothermic, implying that a Sabatier subsystem can simultaneously store chemical energy and provide recoverable heat that may be integrated into upstream/downstream unit operations, improving overall system efficiency (Engelbrecht et al., 2020; Tripodi et al., 2020). This strong exothermicity, however, requires careful thermal management to prevent overheating and loss of catalytic performance (Tommasi et al., 2024).

### 3.6 Water

H_2_O is a central resource for long-duration human missions beyond Earth, as it is required for crew consumption, physicochemical life-support processes, electrolysis-based production of O_2_ and H_2_, and regenerative biological functions including microbial recycling and plant cultivation (Ma et al., 2024; Olawade et al., 2026). Once volatile-bearing regolith or ice-rich material has been accessed through the excavation and drilling strategies discussed above, H_2_O can be obtained primarily through thermal processing, in which the feedstock is heated to induce sublimation or desorption, followed by H2O-vapor capture through condensation or cold-trap systems (Farina et al., 2024; Trautner et al., 2024). H_2_O acquisition in ISRU includes thermal extraction directly from ice-bearing regolith and, in Martian scenarios, recovery from hydrated refractory and volatile materials (Starr and Muscatello, 2020; Inglezakis, 2026). H_2_O recovery in BLSS instead relies on physicochemical purification and recycling of internal waste streams (Pickett et al., 2020; Fahrion et al., 2021; Verbeelen et al., 2021).

Thermal extraction from rocky substrates is physically plausible and supported by laboratory and subsystem demonstrations, but large-scale implementation remains partly speculative until the distribution and concentration of the resource are better constrained (Casanova et al., 2020; Chakraborty et al., 2024; Trautner et al., 2024). Extraction performance is strongly dependent on feed composition and reactor design. In vacuum experiments using an auger-based extraction system, hydrated lunar regolith simulants with an initial H_2_O content of 10 wt% yielded 22.5 g(kW · h)^−1^ after 1 h of heating at 400 W (He et al., 2021). In a microwave-based study on cryogenic icy lunar regolith simulants, H_2_O collection rates of 0.53 g min^−1^-1.79 g min^−1^, collection efficiencies of 29%−63%, and energy costs of 4 Wh g^−1^-7 Wh g^−1^ were reported, with the energy cost decreasing to 2.5 Wh g^−1^ as initial H_2_O content increased from 1.96 wt% to 13.79 wt% (Wang et al., 2026). Thermal extraction is feasible under controlled conditions, but performance is highly sensitive to resource concentration, mineralogical composition, and operating conditions, which complicates direct extrapolation to planetary-scale applications (He et al., 2021; Wang et al., 2026).

At present, the most concrete near-term applications concern the Moon, where orbital observations and impact-based measurements indicate the presence of shallow H_2_O ice and volatile-rich regolith in the polar regions, although the distribution, concentration, and accessibility of these resources remain uncertain (Anand et al., 2014; Biswas et al., 2020; Lucey et al., 2022; Abe, 2024; Li et al., 2025). Mars remains a plausible but more heterogeneous target for H_2_O ISRU, with potential reservoirs including subsurface ice, regolith-bound H_2_O, and atmospheric H_2_O vapor, making the extraction chain more uncertain and strongly dependent on local geological context (Rapp, 2018; Starr and Muscatello, 2020; Inglezakis, 2026).

Current efforts remain focused on prospecting and extraction-enabling operations rather than on establishing sustained production plants. PRIME-1 couples subsurface access with volatile detection. PROSPECT integrates the ProSEED drill, which can acquire cryogenic samples, with the ProSPA analytical package, in which samples are thermally processed to release and characterize volatiles, including H_2_O-bearing phases (Quinn et al., 2023; Trautner et al., 2024).

The main bottleneck, however, is often not the thermal release itself, but the identification of deposits that are sufficiently abundant, shallow, and laterally continuous to justify extraction (Casanova et al., 2020; Chakraborty et al., 2024). Prospecting missions such as LUPEX (Lunar Polar Exploration) are therefore critical because they can help constrain the quantity, quality, distribution, and accessibility of H_2_O and other volatiles in the lunar south polar region (Fisher et al., 2017; Hoshino et al., 2020; Chakraborty et al., 2024; Abe, 2024).

Once recovered, ISRU-derived H_2_O is unlikely to be directly suitable for crew consumption or electrolysis, because co-released contaminants may include dust, salts, metals, sulfur-bearing compounds, and volatile organics. Purification, therefore, represents a distinct downstream step and may involve staged condensation, particulate filtration, adsorption, membrane separation, and other physicochemical polishing processes depending on the intended end use (Pickett et al., 2020; Volpin et al., 2020; Farina et al., 2024). The biological contribution to H_2_O management is potentially significant for purification, recycling, and stabilization of internal H_2_O flows, for example, through LC systems such as MELiSSA, in which wastewater treatment, organic matter conversion, crop cultivation, and transpiration-driven recovery can reduce net H_2_O demand (Verbeelen et al., 2021; Pannico et al., 2022; Frossard et al., 2024).

In MELiSSA-associated hybrid treatment systems, crew-derived wastewater streams representative of one astronaut, comprising 1.2 L d^−1^ urine and 3.4 L d^−1^ shower H_2_O, have been processed through combined biological and physicochemical steps to yield H_2_O meeting ESA hygienic standards, with 87% ± 5% permeate recovery relative to the combined influent (Lindeboom et al., 2020). In the same system, the estimated theoretical primary energy requirement was 0.2 kWhp L^−1^ recovered H_2_O, while the reverse-osmosis stage showed a stable but biofouling-limited permeability of 0.5 L m^−2^ h^−1^ bar^−1^ (Lindeboom et al., 2020). At these recovery rates, such hybrid treatment systems would function as internal H_2_O recovery modules within BLSS, converting crew-derived wastewater into reusable H_2_O and decreasing the make-up H_2_O demand placed on external supply or ISRU-derived reservoirs (Lindeboom et al., 2020; Verbeelen et al., 2021).

### 3.7 Element and waste recycling

#### 3.7.1 Micro-ecological life support system alternative (MELiSSA)

MELiSSA is a long-term research initiative led by ESA, initiated in 1989, with the goal of developing an advanced regenerative LSS for extended space missions. These recycling loops directly condition ISRU feedstock availability (e.g., CO_2_, H_2_O). The project draws inspiration from natural aquatic ecosystems to construct an artificial closed-loop system capable of recycling organic waste, CO_2_, and wastewater into essential resources such as O_2_, H_2_O, and edible biomass (Lasseur et al., 2005; Farges et al., 2008; Lasseur et al., 20810).

MELiSSA is structured into five distinct compartments, each performing a specific ecological function designed to break down, process, and reintegrate waste materials into the system (Lasseur et al., 20810; Walker and Granjou, 2017). The first stage involves the liquefying compartment, where organic waste undergoes anaerobic digestion facilitated by thermophilic bacterial consortia. This process decomposes complex organic compounds into a mixture of ${\text{NH}}_{4}^{+}$, CO_2_, volatile fatty acids, and mineral-rich fluids. The resulting metabolic byproducts are then routed to the photoheterotrophic compartment, where specific families of anoxygenic bacteria, such as *Rhodospirillaceae* and *Rhodobacteraceae*, metabolize the volatile fatty acids while refining the wastewater composition (Hendrickx et al., 2006; Alloul et al., 2019). Subsequently, the nitrifying compartment introduces chemolithotrophic bacteria, i.e., *Nitrosomonas europaea* and *Nitrobacter winogradskyi*, to convert ${\text{NH}}_{4}^{+}$ into ${\text{NO}}_{3}^{-}$ through a controlled two-step oxidation process (Arp and Stein, 2003; Hendrickx et al., 2006) as shown in reactions (R9) and (R10).

Ammonia oxidation (*Nitrosomonas europaea*):

${\text{NH}}_{4}^{\;+}$ + 3/2O_2_ → ${\text{NO}}_{2}^{\;-}$ + 2H^+^ + H_2_O (R9)

Nitrite oxidation (*Nitrobacter winogradskyi*):

$\text{N}{{\text{O}}_{2}}^{-}+0.5{\text{O}}_{2}\to \text{N}{{\text{O}}_{3}}^{-}$ (R10)

Nitrates serve as nutrients for the plant-based components of the system, ensuring sustainable biomass production (Hendrickx et al., 2006; Garcia-Gragera et al., 2021). The fourth stage, the photoautotrophic compartment, is divided into two subcomponents: (i) an algae-based photobioreactor, primarily cultivating *Arthrospira platensis* as a protein-rich food source while concurrently fixing atmospheric CO_2_ and releasing O_2_, and (ii) the higher plant chamber, where crop species such as wheat (*Triticum* spp.), potatoes (*Solanum* spp.), soybeans (*Glycine* spp.), and leafy greens are grown under controlled environmental conditions. In addition to inorganic molecules, this compartment is also able to produce carbohydrates, vitamins, and essential nutrients necessary for sustaining astronauts in space. The final stage of the loop is the crew compartment, where human metabolic activity completes the cycle by consuming O_2_ and food produced while generating exhaled CO_2_ and organic waste, which are reintroduced into the system (Lasseur et al., 20810; Garcia-Gragera et al., 2021). Some reaction processes involved in MELiSSA technology can be grouped in three functional compartments: (i) anaerobic digestion (R11), (ii) nitrification (R12), (R13), and (iii) photosynthesis by cyanobacteria, algae and plants (R1).

C_6_H_12_O_6_ → 3CO_2_+3CH_4_ (R11)

$\text{N}{{\text{H}}_{4}}^{+}+3/2{\text{O}}_{2}\to \text{N}{{\text{O}}_{2}}^{-}+2{\text{H}}^{+}+{\text{H}}_{2}\text{O}$ (R12)

$\text{N}{{\text{O}}_{\text{2}}}^{-}+\text{1}/\text{2}{\text{O}}_{\text{2}}\to \text{N}{{\text{O}}_{\text{3}}}^{-}$ (R13)

The facility has successfully demonstrated sustained microbial activity, plant cultivation under space-like conditions, and efficient nutrient cycling (Lasseur et al., 2005; Garcia-Gragera et al., 2021). Recent advancements in MELiSSA's research have included the development of optimized photobioreactors that enhance the metabolic yield of O_2_-producing microalgae, precision monitoring of microbial consortia stability using genomic and metabolomic techniques, and novel bioregenerative filtration systems capable of recycling urine into drinkable H_2_O with mineral recovery (Zuñiga et al., 2017; Lindeboom et al., 2020; Poughon et al., 2021). MELiSSA faces several challenges that must be addressed before full-scale deployment in space. The optimization of microbial consortia dynamics remains a key research focus, as long-term stability and resistance to spaceflight-induced stressors such as radiation, microgravity, and variable CO_2_ concentrations must be ensured (De Micco et al., 2023). Another critical issue is energy efficiency, as the system relies on controlled temperature, aeration, and light delivery to sustain biological activity. Reducing energy consumption through adaptive lighting for plant growth, waste heat recovery, and autonomous system regulation, or coupling MELiSSA with external ISRU energy-generation systems, will be essential for long-duration missions (Ordoñez et al., 2004; Farges et al., 2008). Advances in wastewater purification, urban aquaponics, and sustainable agricultural practices on Earth have been directly influenced by MELiSSA's closed-loop principles. The knowledge acquired through microbial waste conversion processes has also contributed to the development of biological air filtration systems for industrial applications, as well as carbon capture technologies aimed at mitigating greenhouse gas emissions (Sheoran et al., 2022; Sieborg et al., 2024).

## 4 Linking ISRU and BLSS through coupled architectures

Biological systems can provide capabilities that complement abiotic ISRU, particularly in regeneration, recycling, and multifunctional resource conversion, and *vice versa* (Montague et al., 2012; Verseux et al., 2016; Averesch, 2021; Liu et al., 2021; Gumulya et al., 2022; De Micco et al., 2023). Compared with many physicochemical ISRU processes that rely on high temperatures, elevated energy input, or specialized reactor conditions, biological systems tend to operate under milder conditions, which might reduce their energy requirement (Rawlings and Johnson, 2007; Liu et al., 2021; Verseux et al., 2021; Konieczny et al., 2023) as well as the mass, volume, and complexity of payloads and infrastructure required for space missions (Anand et al., 2012; Heather et al., 2021; Santomartino et al., 2022, 2023; Tonietti et al., 2024). Biological systems can be relatively self-sustaining and adaptive (Scott Jordan and Heidenreich, 2010; Liu, 2020; Corning, 2022). They can replicate, repair molecular damage, and tolerate environmental stress such as radiation, temperature extremes, and nutrient limitation (Pikuta et al., 2007; Zeldovich et al., 2007; Kish and DiRuggiero, 2012; Rampelotto, 2013; Kish et al., 2016; Poli et al., 2017; Di Donato et al., 2018; Merino et al., 2019; Jeong et al., 2024). Where maintenance is constrained, and the supply of resources from Earth is limited, these properties may be particularly advantageous. Biological systems can also be inherently multifunctional (Poli et al., 2017; Di Donato et al., 2019; Finore et al., 2019; Hay Mele et al., 2023; Santomartino et al., 2023; De Stefano et al., 2025). A single biological unit can carry out multiple roles such as producing O_2_, fixing carbon, recycling waste, generating food, and even contributing to structural material synthesis (Ordoñez et al., 2004; Lasseur et al., 20810; Billi et al., 2013; Verseux et al., 2016; Zuñiga et al., 2017; Averesch, 2021; Kong et al., 2021). Abiotic systems, in contrast, tend to be highly specialized, efficient, and locked into a specific function, providing robust and high-throughput extraction but often demanding more hardware, more energy, and tighter process control (Shemer and Semiat, 2017; Cheema and Krewer, 2018; Nasr et al., 2018; Ghavam et al., 2021; Seidel et al., 2022, 2023). Biological systems offer greater regenerative potential and functional integration, but they generally operate more slowly, under narrower environmental tolerances, and with greater sensitivity to instability, contamination, and process-control challenges during scale-up. This multifunctionality could enable the creation of a self-contained, highly interconnected, and circular system that approximates Earth's biosphere at a controllable scale, in which the output from a system becomes the input for another. No abiotic alternative has yet demonstrated the same range of integrated functions (e.g., organic waste recycling, atmospheric regeneration, water recovery, and biosynthesis of complex macromolecules) within a single operational architecture (Mitchell, 1994; Clauwaert et al., 2017; Verbeelen et al., 2021; Nickerson et al., 2022; De Micco et al., 2023).

**Figure 3:**
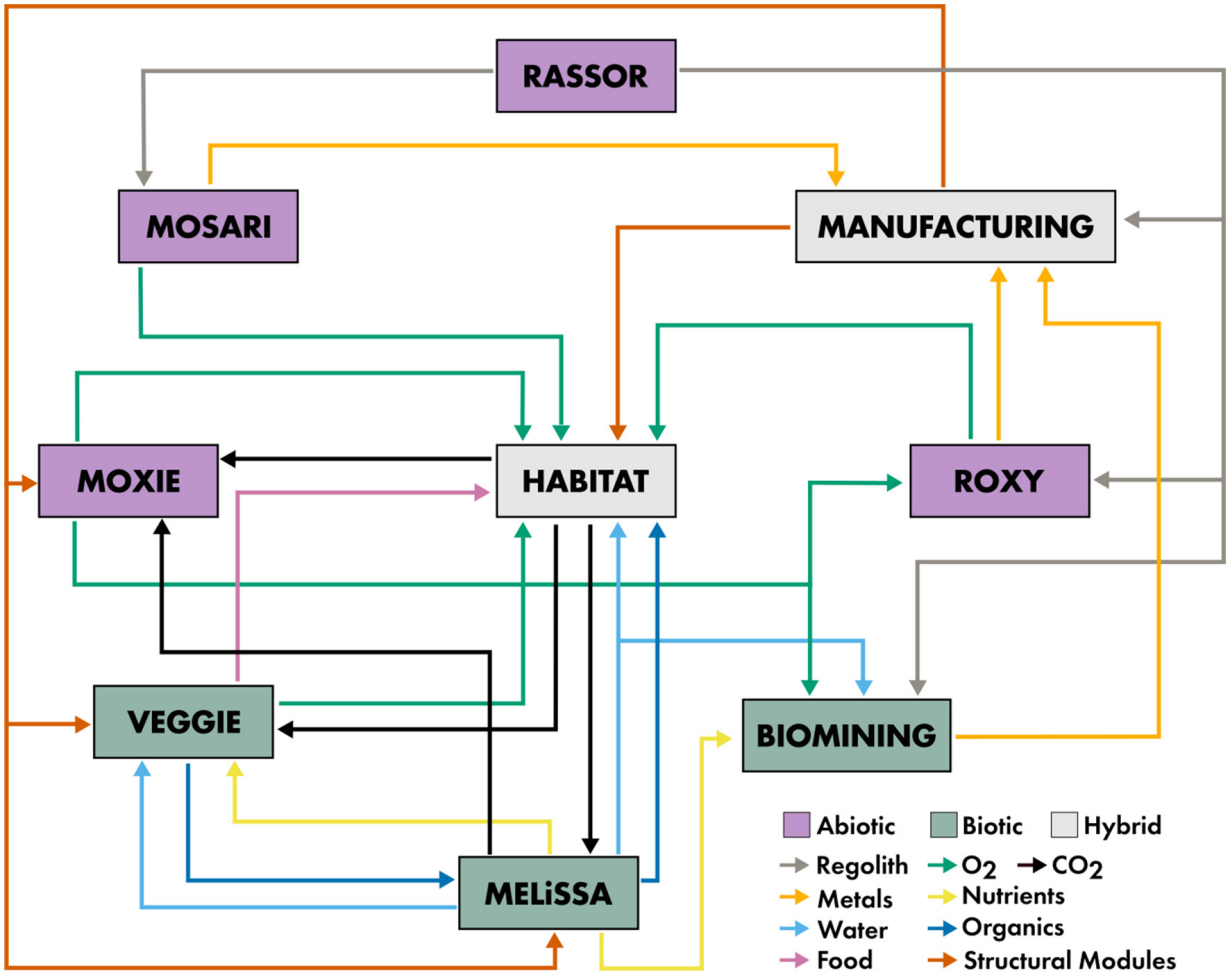
System-level integration of selected functional domains (e.g., biomining, habitat, manufacturing, processing facility) and specific technology demonstrations or mission-relevant projects (e.g., MELiSSA, VEGGIE, MOXIE, ROXY, RASSOR, MOSARI). The figure illustrates interactions among components classified as abiotic (purple rectangles), biotic (green rectangles), and hybrid (gray rectangles). Arrows represent material and energy flows, including regolith (gray), metals (orange), water (light blue), O_2_ (light green), CO_2_ (black), nutrients (yellow), organics (dark blue), food (pink), and structural modules (brown). Project-level elements are shown as representative implementations of the corresponding functional domains, with no assumption of a unique correspondence between projects and functions.

Biology can generate complexity from simplicity. Biotic systems can build highly organized, structured, functional molecules and materials from basic inorganic compounds (Arp and Stein, 2003; Rigoulet et al., 2020; Jiao et al., 2021; Herman et al., 2023). While abiotic methods can produce some of these outputs, they do so at a high energy cost and with far less flexibility. Biological catalysts are highly selective and operate efficiently under mild conditions, even though their intrinsic reaction rates are often lower than those of industrial heterogeneous catalysts used at high temperature and pressure (Wu et al., 2021; Hay Mele et al., 2023; Wang et al., 2023; Farhan et al., 2025). Metabolic pathways often produce very few non-utilizable byproducts and may therefore reduce downstream separation and waste-management burdens (Boodhoo et al., 2022; Lozano and García-Verdugo, 2023). The regenerative and scalable nature of biological systems can provide an important logistical advantage. With local inputs, a small biological system can expand over time into a larger-scale production or processing system. Scalability is fundamental to enable long-term sustainability, independence, and infrastructure development in space (Lasseur et al., 20810; Sanders et al., 2015; Sanders and Kleinhenz, 2022). Biological systems may offer a pathway toward building regenerative habitats able to approximate key functional aspects of terrestrial biogeochemical cycling (Mitchell, 1994; Gòdia et al., 2002; Nelson et al., 2010; De Micco et al., 2023). The integration of biotic and abiotic systems is therefore a strategic step toward architectures that are more efficient, resilient, and adaptive (Figure 3).

From a concept-of-operations perspective, ISRU and LC/BLSS should be treated as distinct but progressively coupled operational modes. ISRU begins with external planetary reservoirs and includes prospecting, excavation or atmospheric capture, extraction, processing, and storage of resources such as H_2_O, O_2_, CO_2_, metals, minerals, and construction feedstocks. LC/BLSS, by contrast, begins with habitat-contained streams and supports the recovery, regeneration, and reuse of air, H_2_O, nutrients, biomass, and organic wastes. Coupling occurs where outputs from one mode become inputs to the other, and is therefore expected to vary with mission class, settlement stage, and planetary context (Averesch et al., 2023). During initial deployment, abiotic ISRU systems will likely dominate prospecting, excavation, power generation, and primary extraction because they provide immediate and robust functionality under resource-limited conditions. As infrastructure expands, BLSS modules can be introduced to support internal LC and extend mission capabilities. In more mature architectures, ISRU-derived products can sustain biological systems, and BLSS can reduce the demand for further extraction by recovering H_2_O, regenerating gases, recycling nutrients, and converting organic waste already circulating within the habitat. Tighter coupling is therefore more likely to characterize long-duration Martian scenarios than early lunar outposts, where abiotic systems are expected to dominate initial deployment and biological modules can be incorporated more progressively as infrastructure and operational complexity increase.

Most space-oriented biological resource-utilization studies still rely on single strains or simplified biological modules, which remain essential for mechanistic testing, process control, and planetary-protection assessment (Cockell et al., 2020; Nangle et al., 2020; Berliner et al., 2022). However, mature resource-processing architectures may ultimately require defined microbial consortia in which complementary organisms distribute functions such as substrate mobilization, redox conversion, organic-waste degradation, nutrient recycling, gas exchange, and product stabilization (Gòdia et al., 2002; Poughon et al., 2021; De Micco et al., 2023). This is particularly relevant where ISRU-derived inputs, such as CO_2_, H_2_O, minerals, or H_2_, must be connected to LC-derived streams, including wastewater, inedible biomass, and organic residues (Averesch et al., 2023). Consortium design should therefore be guided by functional compatibility with mission-relevant feedstocks, target products, environmental constraints, and long-term process stability, with conventional laboratory or industrial model organisms used where they match these requirements (Nangle et al., 2020; Berliner et al., 2022).

A representative of Mars reference architecture can be outlined from the technologies and process classes discussed here. During pre-deployment and early surface setup, abiotic systems would dominate atmospheric processing, H_2_O access, excavation, and primary storage. Atmospheric CO_2_ could feed MOXIE-like units for O_2_ production and storage, and regolith-handling systems analogous to RASSOR or IPEx would support shielding, construction, and the preparation of mineral feedstocks for later processing. Following crew arrival, BLSS modules could be introduced through plant-growth, microbial recycling, and H_2_O-recovery systems, first to supplement habitat support and then to reduce dependence on continued external extraction. As infrastructure matures, ISRU-derived CO_2_, H_2_O, H_2_, O_2_, and mineral resources could supply biological modules for cultivation, microbial conversion, and biomanufacturing. LC/BLSS would recycle H_2_O, regenerate O_2_, recover nutrients, and convert organic residues into biomass or secondary feedstocks, thereby decreasing net resupply and extraction demand. This configuration is intended as a reference architecture assembled from current demonstrators and near-term process classes, not as a deployment-ready system. The individual modules remain heterogeneous in technological maturity and have not yet been demonstrated as integrated surface architecture.

**Figure 4:**
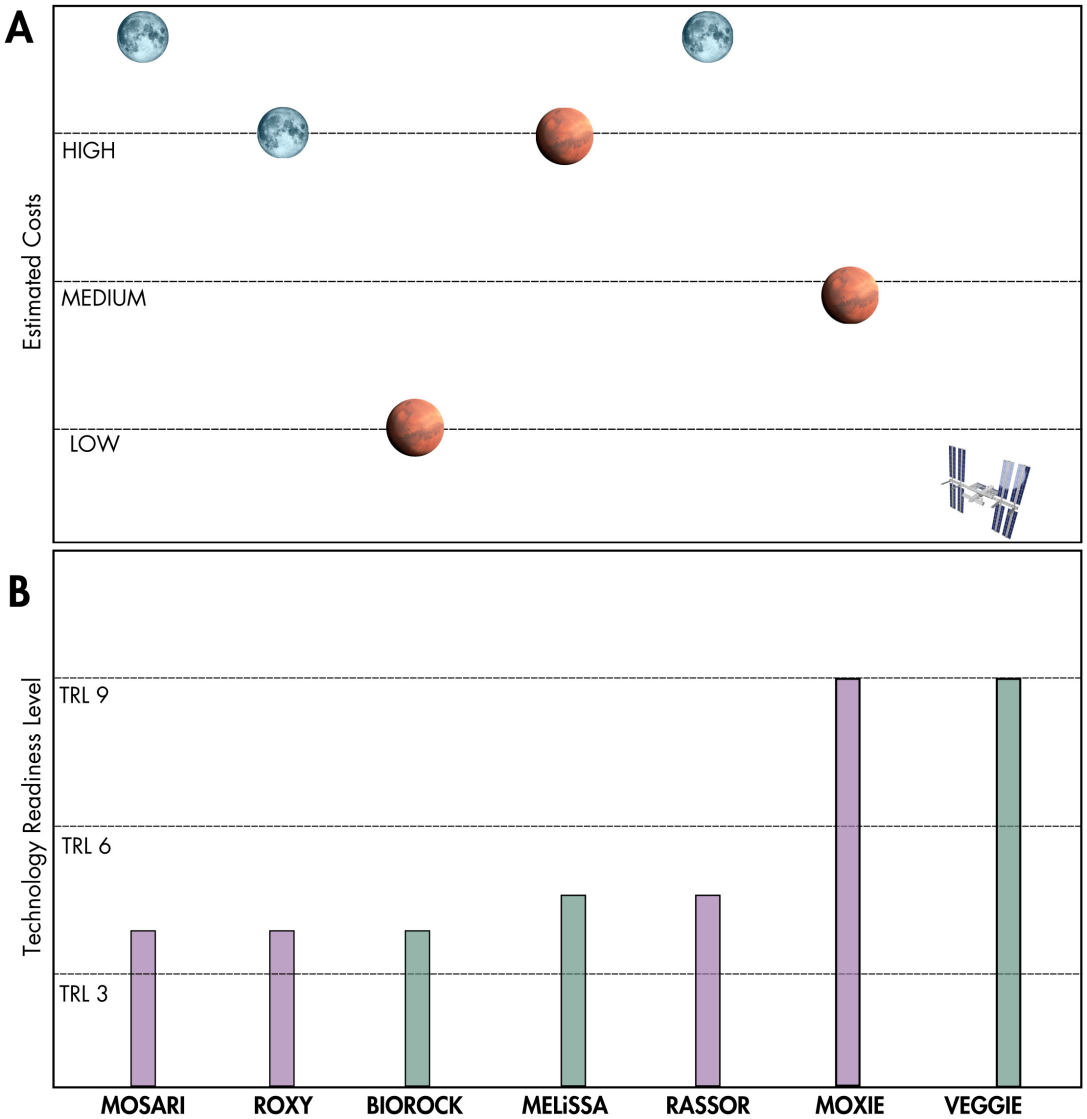
Comparative overview of selected abiotic ISRU technologies (purple bars) and biological life-support or bioprocessing modules relevant to coupled ISRU-BLSS architectures (green bars), shown as a function of primary target location, current relative cost tier **(A)**, and TRL **(B)**. Planetary or spacecraft icons indicate the principal deployment context of each technology (LEO, Moon, Mars), intended as qualitative rather than quantitative estimates. Cost tiers reflect relative system complexity and resource requirements.

A coupled approach could support operation across a broader range of environmental conditions and fluctuations than either type of system alone, while also balancing long-term sustainability with short-term reliability (Jones, 2006; Ellery, 2020). Based on current technical complexity, TRL, and system-integration requirements, we provide a qualitative comparison of relative cost tiers for selected abiotic ISRU technologies and biological modules relevant to these coupled architectures (Figure 4). These relative cost tiers should be interpreted as qualitative system-level comparisons based on complexity, energy demand, and operational infrastructure, rather than as direct economic estimates.

VEGGIE can be placed in the low relative system-complexity tier: it is a simple, lightweight plant-growth system already functional aboard the ISS, using only LED lights and minimal environmental controls that demand little power or hardware (Massa et al., 2020; Bunchek et al., 2021). Biomining, represented in the figure by BioRock-like approaches, can also be considered a low-complexity, low-energy approach, since it relies on microbial processes to mobilize metals from rocks or e-waste under relatively simple reactor configurations (Brandl et al., 2001; Johnson, 2014; Schippers et al., 2014). MOXIE falls within a moderate system-complexity and integration-requirement tier: although it has been tested on Mars, its high-temperature operation and specialized components increase infrastructure and operational demands (Hecht et al., 2021; Hoffman et al., 2022). At the high end, MELiSSA and ROXY are associated with high system-level complexity and infrastructure requirements. MELiSSA's closed-loop life-support system comprises multiple integrated biological and chemical subsystems, and maintaining a stable, closed ecosystem in space increases both development complexity and operational demands (Ordoñez et al., 2004; Lasseur et al., 20810). ROXY operates at 1600 °C and thus requires robust thermal systems, insulation, and substantial power supplies (Seidel et al., 2022, 2023). MOSARI and RASSOR represent the highest expected system-level complexity and infrastructure demand among the reviewed technologies. MOSARI's modular design integrates multiple processes for long-term infrastructure support, demanding advanced automation and significant development resources (Radl et al., 2023). RASSOR must function autonomously in extreme environments, with engineering, mass, and mobility requirements that place its design, testing, and deployment among the most infrastructure-intensive ISRU approaches (Mueller et al., 2013, 2017). Table 2 provides a comparative overview of selected technologies, system demonstrators, and life-support platforms, highlighting differences in maturity, resource flows, operational constraints, inputs and outputs, power demand, scalability, autonomy, and target application. No single approach emerges as optimal across all mission needs. Abiotic systems currently provide the most direct route to high-throughput extraction and conversion, but often at the cost of higher power demand and infrastructure complexity. Biological systems are particularly relevant where regeneration, recycling, and multifunctional processing are needed, although they generally operate more slowly and under narrower environmental constraints. Their coupling may therefore offer the most flexible route toward long-term mission architectures.

**Table 2:** Overview of selected ISRU-related technologies, system demonstrators, and life-support platforms evaluated for Mars, lunar, and ISS applications.

Technologies, Demonstrators, and Projects	TRL (NASA)	Advantages	Disadvantages	Inputs	Output	Power Consumption	Scalability	Autonomy	Target Application	References
MOXIE	9	Demonstrated on Mars; autonomous; scalable in principle	Limited throughput; high-temperature operation; scale-up and sustained operation in dusty Martian environments may increase engineering demands	Martian CO_2_, electricity	O_2_, CO	High	Medium to High	High	Mars atmosphere (O_2_ production)	Hoffman et al., 2015, 2022; Meyen et al., 2016; Hecht et al., 2021; McClean et al., 2020
ROXY	3–4	Dual O_2_/metal production; no H_2_ required; potentially scalable	>1600 °C; reactor wear; material constraints	Regolith, electricity, molten salts	O_2_, Fe, Ti, Al	High	High	Medium	Lunar/Martian regolith	Seidel et al., 2022, 2023; Birch et al., 2025, 2026; Schwandt et al., 2012
MOSARI	3–4	Produces alloys + O_2_; reduces waste; efficient material use	Needs fluorides; requires initial electrolysis	Regolith, electricity, and fluoride salts	O_2_, Ca/Mg/Al/Si alloys	High	Medium	Medium	Lunar regolith (construction metals)	Radl et al., 2023; Vogel et al., 2017; Milicevic et al., 2018; Cvetković et al., 2020
RASSOR	4–5	Prototype excavation under regolith-analog conditions; modular; pre-tested	Small payloads; abrasive dust wear	Mechanical input, regolith	Transported regolith	Medium	High	High	Lunar/Martian soil collection	Mueller et al., 2013, 2017; Radl et al., 2023
Space Biomining (process concept)	3–4	Low energy; selective metal extraction; proven on Earth, but still experimental for space ISRU	Slow kinetics; microbe sensitivity; input variability	Regolith, microbes, nutrients, H_2_O	Dissolved metals (e.g., Fe, Mn, Cu)	Low	High	Medium	Lunar/Martian regolith, meteorites	Anand et al., 2012; Cockell et al., 2021; Kaksonen et al., 2021; Roberto and Schippers, 2022; Guerrero-Gonzalez and Zabel, 2023; Santomartino et al., 2023
MELiSSA	4–5	Closed-loop BLSS architecture; O_2_, water, food, and waste recycling	Complex, high-energy demand; integration challenges	Organic waste, CO_2_, H_2_O, light	O_2_, clean water, food biomass	High	Medium	Medium	Long-term missions (closed-loop life support)	Lasseur et al., 2005; Farges et al., 2008; Lasseur et al., 20810
VEGGIE	9	Fresh food; O_2_ recycling; morale boost	Low food yield; microgravity challenges	Seeds, H_2_O, nutrients, CO_2_, light	Edible crops, O_2_	Medium	Low to Medium	Medium	ISS, Lunar/Mars greenhouses	Massa et al., 2016, 2020; Ehrlich et al., 2017; Bunchek et al., 2021

Approaches are compared across key metrics including NASA TRL, primary advantages and disadvantages, required inputs and generated outputs, power consumption, scalability, degree of autonomy, target application, and representative references.

Comparable system-level considerations also apply to the remaining technologies discussed in this review, including IPEx, TRIDENT, FSP, PlanetVac, UltraFlex, APH, LADA, and PROSPECT (Mason et al., 2008; Paulsen et al., 2018; Schuler et al., 2024; Zacny et al., 2024; Cloud et al., 2025). Although these systems are not discussed individually in the qualitative tiering above, they can be interpreted within the same framework of maturity, infrastructure demand, autonomy, and coupling potential summarized in Table 2.

However, the deployment of biological modules must comply with COSPAR planetary protection policies to avoid compromising life-detection experiments and forward contamination risks (Olsson-Francis et al., 2023).

## 5 Conclusion

We examined abiotic, biotic, and coupled resource-acquisition and regeneration pathways within a resource-oriented framework for sustained human presence beyond LEO. ISRU and LC/BLSS were treated as operationally distinct but architecturally connected systems: ISRU acquires and converts external planetary resources, while LC/BLSS recovers, regenerates, and transforms resources circulating within the habitat. Maintaining this distinction is important because external resource acquisition and internal LC operate on different reservoirs and constraints. In long-duration missions, these functions must still be coordinated so that ISRU-derived products can support habitat operations and LC/BLSS can reduce further extraction demand.

The catalog developed here indicates complementary roles for abiotic and biological systems. Abiotic processes currently provide the most mature routes for high-throughput extraction and conversion, including O_2_ production, H_2_O extraction, regolith processing, and propellant-relevant chemistry. Biological systems add regenerative and multifunctional capacity through waste stabilization, atmospheric revitalization, food production, selective bioprocessing, and biomanufacturing. Defined microbial consortia may further improve the processing of heterogeneous ISRU and LC-derived streams, provided that organism selection is guided by feedstock compatibility, target products, environmental constraints, and process stability.

Future work should move from isolated pathway demonstrations to integrated resource-flow experiments. H_2_O extraction, purification, electrolysis, O_2_/H_2_ storage, biological use, wastewater recovery, and reintegration into LC should be tested as connected processes. Similar integration is needed for CO_2_ capture, H_2_ generation, CH_4_ synthesis, biomass production, biopolymer formation, and organic-waste recycling. These experiments should be accompanied by techno-economic, mass-balance, and life-support demand analyses that define the quantities of O_2_, H_2_O, C, H_2_, CH_4_, nutrients, materials, and energy required for specific crew sizes, mission durations, and planetary settings. Such quantitative framing would help identify which combinations of ISRU and BLSS modules are realistic for early lunar missions, sustained lunar infrastructure, Mars transit, and long-duration Martian settlements.

Sustainable space exploration will require a shift from technology-by-technology validation toward mission-specific resource architectures. A resource-acquisition catalog is useful because it shows which pathways can be connected, which interfaces remain limiting, and how abiotic and biological processes can be coordinated within quantitatively constrained mission systems. The incorporation of biological processes can extend resource utilization beyond extraction, supporting a more regenerative and circular infrastructure that can be managed as a controlled technological ecosystem.
